# Mir-34a Is Upregulated during Liver Regeneration in Rats and Is Associated with the Suppression of Hepatocyte Proliferation

**DOI:** 10.1371/journal.pone.0020238

**Published:** 2011-05-31

**Authors:** Huan Chen, Yimin Sun, Ruiqi Dong, Shengsheng Yang, Chuanyong Pan, Dao Xiang, Mingyong Miao, Binghua Jiao

**Affiliations:** 1 Department of Biochemistry and Molecular Biology, Second Military Medical University, Shanghai, China; 2 National Engineering Research Center for Beijing Biochip Technology, Beijing, China; 3 Department of Cellular Biology, Second Military Medical University, Shanghai, China; French National Center for Scientific Research - Institut de biologie moléculaire et cellulaire, France

## Abstract

**Background:**

MicroRNAs are a class of small regulatory RNAs that modulate a variety of biological processes, including cellular differentiation, apoptosis, metabolism and proliferation. This study aims to explore the effect of miR-34a in hepatocyte proliferation and its potential role in liver regeneration termination.

**Methodology/Principal Finding:**

MiR-34a was highly induced after partial hepatectomy. Overexpression of miR-34a in BRL-3A cells could significantly inhibit cell proliferation and down-regulate the expression of inhibin βB (INHBB) and Met. In BRL-3A cells, INHBB was identified as a direct target of miR-34a by luciferase reporter assay. More importantly, INHBB siRNA significantly repressed cell proliferation. A decrease of INHBB and Met was detected in regenerating liver.

**Conclusion/Significance:**

MiR-34a expression was upregulated during the late phase of liver regeneration. MiR-34a-mediated regulation of INHBB and Met may collectively contribute to the suppression of hepatocyte proliferation.

## Introduction

The liver has a remarkable capacity to regenerate itself in response to signals as physical, chemical, nutritional, vascular, or virus-induced liver injury [1]. Partial hepatectomy (PHx) is widely used as a liver regeneration (LR) model in scientific research since it is free of side effects associated with toxic regenerative stimuli [2]. LR after PHx can be divided into three distinct phases: an initiation step, a proliferation step and a termination step [3]–[5]. In the termination stage, the newly divided cells may exit from the cell cycle under the regulation of some ‘stop’ signals, and return to the G0 quiescent state [6]. However, these ‘stop’ signals leading to cell proliferation inhibition are still poorly defined.

Transforming growth factor-β is the most well-recognized candidate for the ‘stop’ signal [7], [8], because it is highly expressed in the late phase of liver regeneration and can strongly inhibit hepatocyte proliferation *in vitro* and *in vivo*. Another potential candidate is activin, which belongs to the TGF-β superfamily. Activin A, a homodimer of two β A subunits encoded by the inhibin βA (*INHBA*) gene, is an autocrine inhibitor of hepatocyte DNA synthesis and is strongly increased at 3–5 d after PHx [9], [10]. When the action of activin A is neutralized by administration of follistatin, an activin antagonist, liver regeneration after partial hepatectomy is accelerated [11], [12]. Activin B, a homodimer of two βB subunits encoded by the inhibin βB (*INHBB*) gene, is related to activin A. However, it still remains unknown whether activin B has any roles in hepatocyte proliferation. Moreover, a recent study have shown that integrin-linked kinase (ILK) may also play an important role in controlling the termination of LR, partly through HGF/Met, β-catenin and Hippo kinase pathways [13]. Thus, as potential regulators in the termination stage of LR, the specific roles and mechanisms of these factors remain to be elucidated.

MicroRNAs (miRNAs) are a class of small regulatory RNAs that modulate a variety of biological processes, including cellular differentiation, apoptosis, metabolism and proliferation, by targeting different genes [14]. Recently, some studies have described the roles of miRNAs in the process of LR. For instance, miR-21 expression was up-regulated during the early phases of LR, which inhibits Peli1 and potentially regulate NF-κB signaling [15]; miR-23b was down-regulated in the termination phase of LR, and may contribute to activation of the TGF-β1/Smad3 signaling [16]. Thus, analysis about microRNA and related target genes may provide unique insights into the ‘stop’ signal of LR and hepatocyte proliferation.

In the present study, we mainly focused on miR-34a based on its expression pattern after PHx and its antiproliferative function in rat hepatocytes, along with its target genes. Our data suggests that miR-34a might also be a potential ‘stop’ signal that contributes to the suppression of hepatocyte proliferation during the late phase of LR.

## Results

### MiR-34a is induced in response to PHx

**Figure 1:**
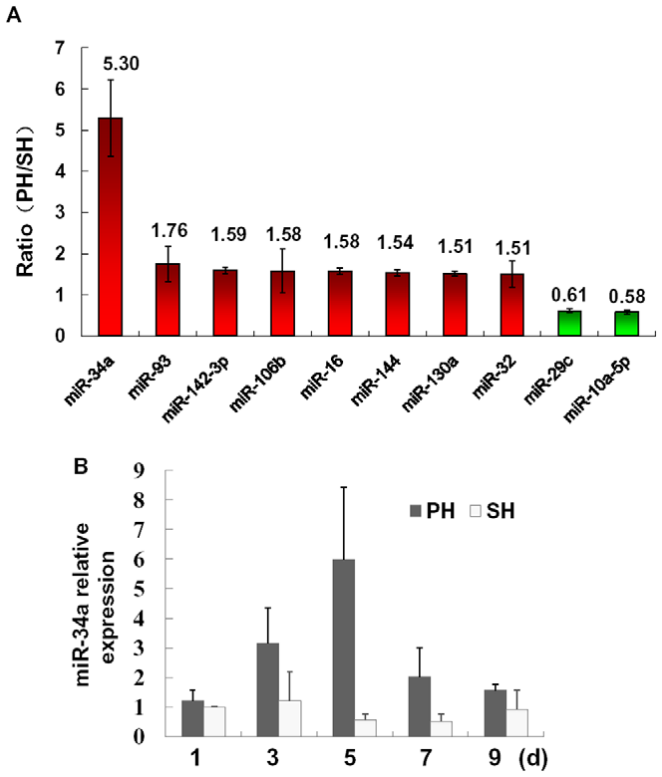
Differentially expressed miRNAs during liver regeneration (LR). (**A**) miRNA expression profiling at 5 d after partial hepatectomy (PHx). Each data point represents the ratio of miRNA expression levels under PHx to Sham operation (SH). Ratio values >1.5 or <0.7 were considered up-regulated (red) or down-regulated (green) in PHx rats compared to SH rats. (**B**) The expression pattern of miR-34a during LR by quantitative real-time PCR (qRT-PCR) analysis. miR-34a levels were normalized to that of *u6*. * *P*<0.05, ** *P*<0.01, vs SH control at each time point.

To illustrate the miRNA expression pattern in the termination phase of LR, we performed a comprehensive miRNA expression profiling analysis on 6 rats 5 d after PHx or SH. As shown in Figure 1A, we identified 8 up-regulated and 2 down-regulated miRNAs, in which miR-34a had at least 5-fold difference in expression between PHx and SH, with a *P* value <0.01. We then studied how miR-34a expression changes during LR on different time points. As revealed by qRT-PCR analysis, the regeneration of the remnant livers after PHx caused a transient increase in miR-34a expression with ∼2-fold and ∼6-fold at 3 and 5 d when compared with SH control on each time point (Figure 1B).

### MiR-34a inhibits BRL-3A cell proliferation

**Figure 2:**
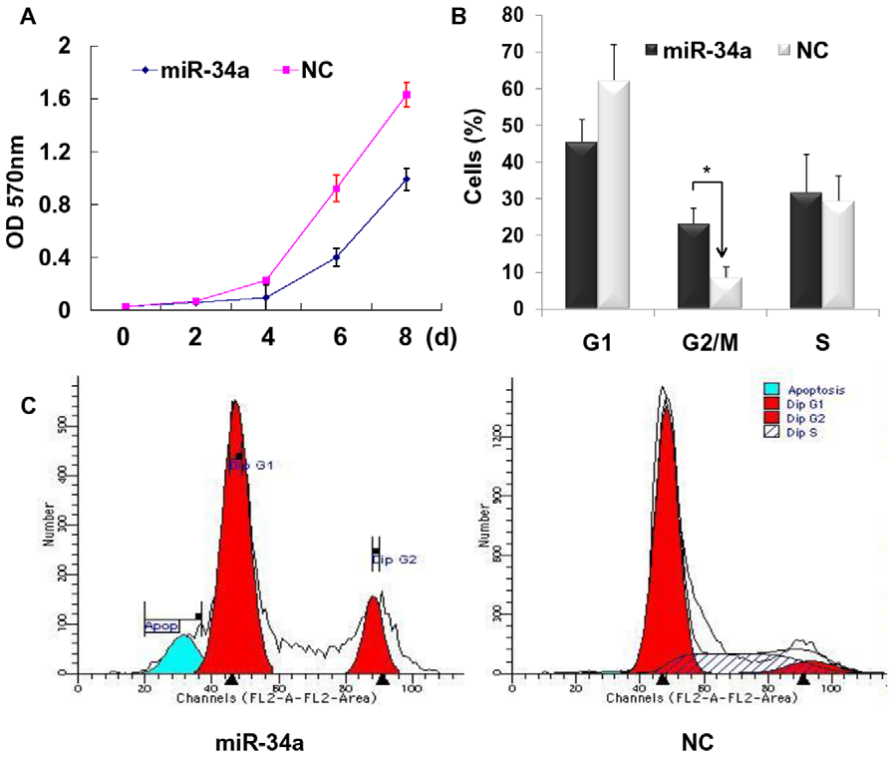
MiR-34a induced growth inhibition in rat hepatocytes. (**A**) BRL-3A cells treated with miR-34a mimics (miR-34a) or negative control (NC) were seeded in 96-well plates and examined at indicated time points. The absorbance of methylthiazoletetrazolium by each sample was recorded at 570 nm after staining. (**B,C**) Cells treated with miR-34a or NC were analyzed by flow cytometry as described in “Materials and methods” for cell cycle distribution analysis.

To evaluate the effect of miR-34a in regulating rat liver cell proliferation, a MTT cell proliferation assay and a cell cycle analysis were used (described in “Materials and methods”). Briefly, BRL-3A immortalized rat liver cells were transfected with miR-34a or NC. MTT assay was performed at 2-day intervals, while cell cycle analysis was conducted 48 h after infection. As shown in Figure 2A, miR-34a markedly reduced BRL-3A cell growth at 4 d and more remarkably at 6 d (*P*<0.01). The growth inhibitory effect of miR-34a can also be sustained by the data of cell cycle analysis, in which the percentages of G2 phase cells in miR-34a and NC groups were (23.14±4.26)% and (8.48±2.93)% respectively, indicating a subpopulation of cells arrested in G2 phase by miR-34a (Figure 2B,C).

### MiR-34a down-regulates INHBB and Met expression

Although the precise mechanisms of the termination of LR remain elusive, some important pathways have been linked to this process, such as, TGF-β, activin A, and ILK pathways [7]–[9], [13]. Therefore, we presumed that miR-34a may possess some target genes associated with these pathways. Bioinformatic tools for putative miR-34a target genes (Targetscan, http://www.targetscan.org/) and pathway classification analysis of these candidate target genes (Molecular Annotation System, MAS, http://bioinfo.capitalbio.com/mas) were used (Tables S1, S2).

**Figure 3:**
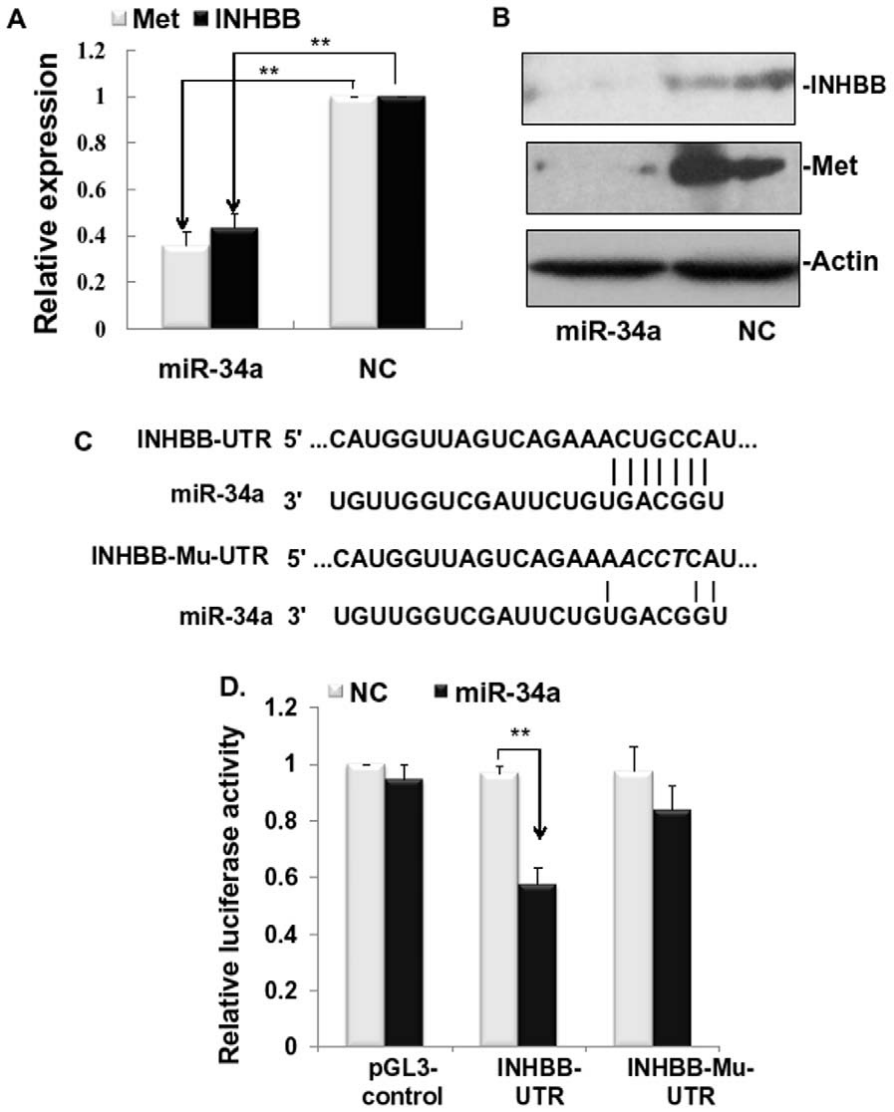
Analyses of candidate target genes of miR-34a. (**A**) miR-34a decreased mRNA expression of inhibin beta B (*INHBB*) and *Met* by qRT-PCR. (**B**) miR-34a decreased protein expression of INHBB and Met by westernblot analysis. Actin was used as sample control. (**C**) miR-34a-binding site in the 3′-UTR (top) and mutated sites in 3′-UTR (bottom) of *INHBB* constructed in a luciferase system. (**D**) The 3′-UTR of *INHBB* mediates *INHBB* repression. BRL-3A cells were co-transfected with a luciferase reporter vector containing the 3′-UTR or mutated sequence of *INHBB* and miR-34a mimics (miR-34a) or negative control (NC). pGL3-control vector was used as control. **P<0.01 vs NC.

*INHBB*, which encodes a subunit of activin AB and activin B, was identified by bioinformatic analysis. Although Met has been proved to be a target of miR-34a in HeLa cells and HepG2 cells [17], [18], it is still involved in validation in BRL-3A cells. qRT-PCR and westernblot analysis revealed that miR-34a drastically inhibited the expression of INHBB and Met on both mRNA and protein levels (Figure 3A,B). To detect whether the regulation of INHBB was direct, we fused the 3′-UTR region of *INHBB* to a luciferase system. As shown in Figure 3C,D, miR-34a remarkably repressed the expression of luciferase containing an original miR-34a binding site (*INHBB*-UTR) but not a mutant binding site (*INHBB*-Mu-UTR). And mutations in seed complementary sites of the 3′-UTR region of *INHBB* could restore the luciferase expression.

### INHBB downregulation represses hepatocyte proliferation

**Figure 4:**
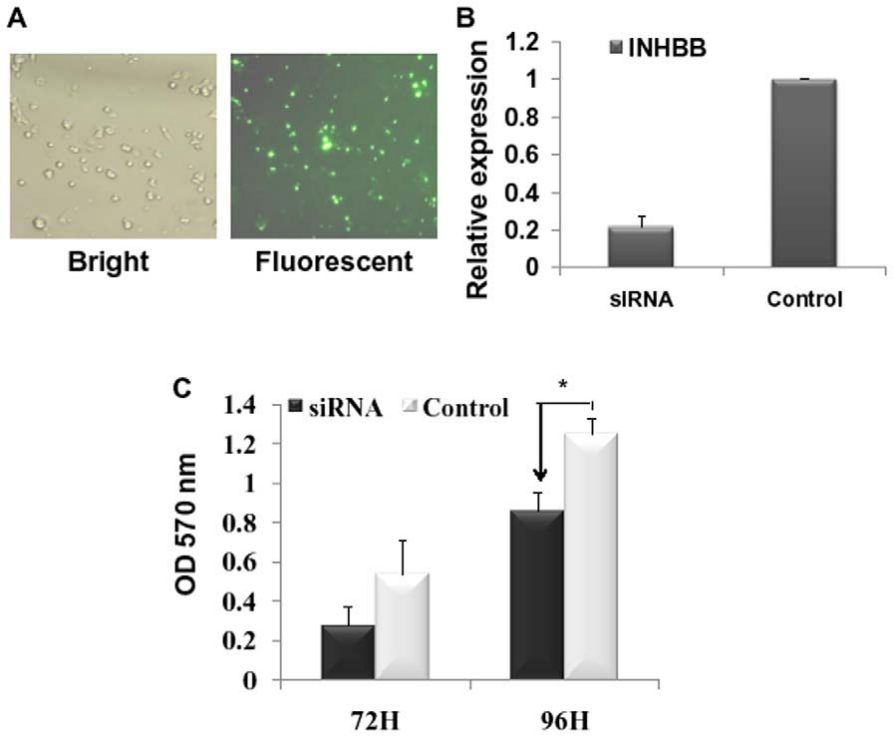
INHBB downregulation repressed hepatocyte proliferation. (**A**) Fluorescence (right) and bright light (left) photomicrographs of FAM-positive cells transfected by FAM-siRNA. Pictures were taken 6 h after transfection. (**B**) Identification of INHBB knockdown efficiency by siRNA via qRT-PCR. Actin was used as fold control. (**C**) INHBB silencing led to growth inhibition in BRL-3A cells by MTT cell proliferation analysis. (siRNA: INHBB siRNA; Control: control siRNA) *P<0.05 compared to control.

In the present study, *Met* and *INHBB* were confirmed as the target gene of miR-34a. Met is known to play a critical role in the progressing and termination stage of liver regeneration [13]. However, the function of INHBB in hepatocyte proliferation still needs to be clarified. In order to prove that INHBB functions as a promoter in proliferation like Met, we then studied the effect of INHBB siRNA on hepatocyte proliferation. Cells were transfected with INHBB siRNA, Control siRNA or FAM-siRNA Control. The FAM-siRNA Control enables visualization of transfection efficiency 4–6 hours post transfection via bright (Figure 4A, left) and fluorescence (Figure 4A, right) microscope. The knockdown efficiency of INHBB was verified by real-time PCR (Figure 4B). In MTT cell proliferation assay, cells treated with INHBB siRNA or Control siRNA sequences were re-seeded in 96-well plates 48 h post transfection (described in “Materials and methods”) and were allowed to grow for indicated times. As revealed in Figure 4C, INHBB siRNA strongly repressed BRL-3A cell proliferation (*P*<0.05).

### Expression of miR-34a candidate target genes in regenerating liver tissues

**Figure 5:**
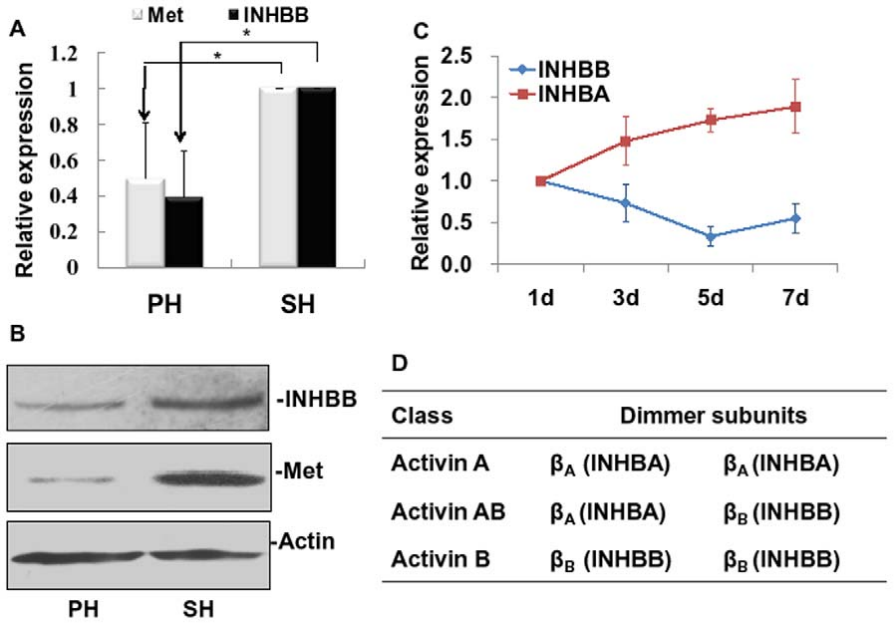
Expression of INHBB and Met in regenerating livers. (**A**) mRNA expression of *INHBB* and *Met* in regenerating liver at 5 d after resection by qRT-PCR. (**B**) Protein expression of INHBB and Met in regenerating liver at 5 d by westernblot analysis. Actin was used as sample control. (**C**) Expressive patterns of *INHBB* and *INHBA* mRNA in the regenerating liver after PHx by qRT-PCR. (**D**) The activin protein complexes are composed of inhibin beta A (INHBA) and inhibin beta B (INHBB) subunits.

To determine whether miR-34a potentially regulates the expression of INHBB and Met during LR, we tested the expression of INHBB and Met in regenerating liver tissues at 5 d after PHx, when miR-34a was highly induced. By qRT-PCR and westernblot analysis, we observed intensive down-regulation of INHBB and Met on both mRNA and protein levels in PHx rats, indicating that miR-34a and its two candidate target genes were inversely expressed (Figure 5A,B). Moreover, we found that after PHx *INHBB* mRNA had declined at 3 d, and was markedly repressed at 5 d and 7 d; while *INHBA* mRNA had increased at 3 d, and then was strongly increased at 5 d and 7 d (Figure 5C). Our data indicated that activin B (homodimer of two INHBB proteins) had an opposite expression pattern as compared to activin A (homodimer of two INHBA proteins) (Figure 5D).

## Discussion

Despite strong evidence that miRNAs are involved in the priming and progression phase of LR, little is known about how miRNAs affect the termination stage. To gain more insights into the roles of miRNAs, we performed a miRNA microarray analysis on late-phase regenerating livers. MiR-34a with ∼6-fold change was identified by microarray and qRT-PCR (Figure 1). Intriguingly, miR-34a is well known for its anti-oncogenic activity in several cancers, including hepatocellular carcinoma [17], [19]–[21]. Therefore, we hypothesized that miR-34a was a key suppressor of hepatocyte proliferation and might be a negative regulator during LR, like other ‘stop’ signals as TGF-β and activins.

To confirm the role of miR-34a in normal hepatocytes, we used cultured rat liver cells (BRL-3A cells) as cell models. Our data showed that miR-34a drastically inhibited BRL-3A cell growth and induced a significant G2/M arrest (Figure 2). However, in previous study, ectopic miR-34a was shown to induce a cell cycle arrest in the G1-phase, thereby suppressing tumor cell proliferation [18], [22], [23]. We assumed that the discrepancy may be caused by the different backgrounds and molecular mechanisms between normal cells and tumor cells. For instance, in tumor cells, the aberrant activated E2F complexes can work on the target genes whose products regulate the G1/S transition; and loss of regulation at the G1/S transition appears to be a common event among virtually all types of human tumor [24]. Moreover, our data were also supported by a recent study of Factor, who discovered that c-Met deficient hepatocytes were blocked in early/mid G2 phase [25].

To determine how miR-34a influences the hepatocyte proliferation, we then used a Molecular Annotation System (MAS) to categorize all putative target genes of miR-34a predicted by Targetscan (Table S1). Interestingly, we identified INHBB as a target gene of miR-34a in the activin pathway. In this study, we show that miR-34a not only inhibits INHBB in a direct way (Figure 3) but also may result in the down-regulation of INHBB in regenerating liver (Figure 5). More importantly, we proved that knockdown of INHBB via a siRNA system could strongly repress rat hepatocyte proliferation (Figure 4). In activin family (Figure 5D), activin A (homodimer of two INHBA proteins) has been shown to decelerate hepatocyte growth in LR. Interestingly, in our findings, activin B (homodimer of two INHBB proteins) seemed to play an opposite role in cell proliferation with an opposite mRNA expression pattern after PHx (Figure 5C). There have been a few reports comparing the biological potency of activin A and activin B. For example, stimulation of DNA synthesis by EGF could be inhibited by activin A, but not by activin B [26]. It has been reported that activin A and activin B had opposite effects on Ca^2+^ signaling in islet cells, with activin A increasing, but activin B decreasing [27]. Therefore, it is conceivable that the overall effect of activins during LR may result from the balance between the expression of *INHBB* and *INHBA* subunit genes.

Apart from INHBB, we also confirmed Met as another target of miR-34a in the regenerating livers. It has been reported that an increase in Met, together with its ligand HGF, could lead to impaired liver regeneration [13]. In accordance with previous study, our investigation suggests that miR-34a-mediated inhibition of Met may also contribute to the suppression of hepatocyte proliferation during LR.

In conclusion, miR-34a is strongly induced in the late phase of LR after PHx. Elevated miR-34a greatly suppressed hepatocyte proliferation by targeting INHBB and Met. Our data also provided a tantalizing hint that miR-34a might be a ‘stop’ signal in regenerating hepatocytes.

## Materials and Methods

### Ethics statement

All animals were cared appropriately according to the Institutional Animal Care Instructions approved by the Animal and Ethics Review Committee, Second Military Medical University, Shanghai, China. All experimental procedures were approved by the Animal and Ethics Review Committee of the Second Military Medical University (SCXK 2007-0003).

### Animals and operative procedure

Male Sprague-Dawley rats (180–210 g) were maintained on a normal diet with free access to water. After they were anesthetized with sodium pentobarbital (50 mg/kg, intraperitoneally), 70% partial hepatectomy (PHx) was performed as described by Higgins and Anderson [28]. For sham operation (SH), rats underwent abdominal surgery without liver resection. At indicated time points: 1, 3, 5, 7 days (d) after surgery, animals were sacrificed and the remnant liver tissues were collected.

### Microarrays

Total RNA samples from SH and PHx rats (5 d after PHx) were analyzed by CapitalBio Corporation (CapitalBio, Beijing, China) for miRNA microarray experiments as described before [29]. Briefly, miRNA was separated from 20–30 µg total RNA using the Ambion miRNA Isolation Kit (including small RNAs). Fluorescein-labeled miRNAs were used for hybridization on each miRNA microarray chip containing 1320 probes in triplicate, corresponding to 988 human (including 122 predicted miRNAs), 627mouse, and 350 rat miRNAs found in the miRNA Registry (http://microrna.sanger.ac.uk/sequences/). The differential miRNAs were selected using the program Significance Analysis of Microarrays (version 2.1). The alterations were defined as those with either <0.7- or >1.5-fold changes and the d value <0.05.

### Reverse transcription and quantitative real-time PCR

Total RNA was isolated from prepared liver samples or transfected cells by Trizol (Invitrogen, Carlsbad) reagent. cDNA was synthesized following the manufacturer's protocol (MBI Fermantas). Quantitative real-time PCR (qRT-PCR) was performed using a standard SYBR green PCR kit (TOYOBO), and PCR-specific amplification was applied in the Applied Biosystems (ABI7300) real-time PCR machine. The relative expression of target genes (miR-34a, *u6*, *INHBB*, *Met*, *INHBA* and *Actin*) was calculated with the 2^−△△^Ct method [30]. Primers are listed (Table S3).

### Cell culture and transient transfection

An established rat liver cell line BRL-3A was obtained from the Institute of Biochemistry and Cell Biology, Shanghai, China. BRL-3A cells were grown in RPMI1640 (GIBCO) with 10% fetal bovine serum (FBS; GIBCO) in a humidified atmosphere containing 5% CO_2_ at 37°C. MiR-34a mimics, negative control sequences, INHBB siRNA and control siRNA were obtained from GenePharma, Shanghai, China. All sequences are listed in Table S1. Transfections were performed using a Lipofectamine™ 2000 kit (Invitrogen) according to the manufacturer's instructions. To monitor the transfection efficiency, Fluorescein (FAM)-siRNA (GenePharma) was used as control. And successfully transfected cells were observed under a fluorescence microscope.

### Cell proliferation assay by methylthiazoletetrazolium

BRL-3A cells transfected with miR-34a mimics (miR-34a) or negative control (NC) (GenePharma) and cells transfected with INHBB siRNA or control siRNA in the 24-well plate were re-seeded in 96-well plates at an optimized density cells 48 hours (h) after transfection. At indicated time points, 20 µL methylthiazoletetrazolium (MTT) solution (5 mg/mL) was added into the culture medium for 4 h incubation. Then 150 µL dimethyl sulfoxide (DMSO) was added to each well to dissolve the crystals. The absorbance of each sample was recorded at 570 nm after 10 minutes.

### Cell cycle analysis by flow cytometry

BRL-3A cells were cultured in 6-well plates and then treated with miR-34a or NC. 48 h later, cells were collected, fixed by 70% ethanol for 30 min and then washed with ice-cold PBS. The cell pellets were re-suspended in RNase-containing (1∶100 in dilution) PBS buffer on ice. At last, the cells were stained with propidium iodide (PI) and then analyzed using a flow cytometer.

### Plasmid construction and Luciferase reporter assay

The 3′-UTR of *INHBB* containing the *INHBB*-miR-34a response element was cloned into the *Xho*I/*Fse*I site of pGL3 control Luciferase vector (Promega). A mutant 3′- UTR of *INHBB* was synthesized by PCR. Primers are listed in Table S1. BRL-3A cells were seeded in a 24-well plate (1×10^5^ per well) and transfected with *INHBB*-UTR-pGL3/Mu-INHBB-UTR-pGL3 (200 ng), Renilla luciferase control vector (20 ng) and miR-34a/NC (15 pmol). 48 h later, all protein extracts were analyzed using the dual luciferase reporter assay system (Promega).

### Westernblot analysis

The levels of INHBB, Met and Actin were determined in whole-cell extracts from liver tissues (5 d after PHx or SH) or transfected cells (miR-34a or NC). Protein extracts were separated on a SDS–polyacrylamide gel, blotted onto a nitrocellulose membrane (Millipore), and incubated with anti-INHBB (Abcam), anti-Met (Cell Signaling) or anti-Actin (Cell Signaling). Immunoblots were developed using goat anti-rabbit or anti-mouse secondary antibody, followed by detection with enhanced chemiluminescence (Pierce).

### Statistical analysis

The data shown are represented as means ± standard deviation. A *P*-value of less than 0.05 was considered statistically significant.

## Supporting Information

Table S1 **Candidate genes of rno-miR-34a, predicted by TargetScan. **(Release 5.1: April 2009, http://www.targetscan.org/).
(DOC)

Table S2 **Pathways analysis of miR-34a candidate genes using MAS software (** http://bioinfo.capitalbio.com/mas **).** Three pathways related to the termination stage of liver regeneration are listed.
(DOCX)

Table S3 **Primers and sequences for qRT-PCR, siRNA transfection and plasmids construction.**
(DOCX)
