# Supplementary material for: Mir-34a Is Upregulated during Liver Regeneration in Rats and Is Associated with the Suppression of Hepatocyte Proliferation
Source: PLoS One. 2011 May 31;6(5):e20238. doi: 10.1371/journal.pone.0020238 (PMC3105003; doi:10.1371/journal.pone.0020238)
Supplement: Table S1 — Candidate genes of rno-miR-34a, predicted by TargetScan. (Release 5.1: April 2009, http://www.targetscan.org/). (DOC) [file pone.0020238.s001.doc]

**Table S1.**

| **Human ortholog of target gene** | **Gene name** | **Total context score** | **Previous TargetScan publication(s)** |
| --- | --- | --- | --- |
|
| [HCN3](http://www.ncbi.nlm.nih.gov/sites/entrez?Db=gene&Cmd=ShowDetailView&TermToSearch=57657) | hyperpolarization activated cyclic nucleotide-gated potassium channel 3 | -1.38 | 2005, 2007 |
| [TTC19](http://www.ncbi.nlm.nih.gov/sites/entrez?Db=gene&Cmd=ShowDetailView&TermToSearch=54902) | tetratricopeptide repeat domain 19 | -0.81 | 2005, 2007 |
| [SATB2](http://www.ncbi.nlm.nih.gov/sites/entrez?Db=gene&Cmd=ShowDetailView&TermToSearch=23314) | SATB homeobox 2 | -0.78 | 2005, 2007 |
| [GREM2](http://www.ncbi.nlm.nih.gov/sites/entrez?Db=gene&Cmd=ShowDetailView&TermToSearch=64388) | gremlin 2, cysteine knot superfamily, homolog (Xenopus laevis) | -0.77 | 2005, 2007 |
| [LBA1](http://www.ncbi.nlm.nih.gov/sites/entrez?Db=gene&Cmd=ShowDetailView&TermToSearch=9881) | lupus brain antigen 1 | -0.75 |  |
| [PPP1R11](http://www.ncbi.nlm.nih.gov/sites/entrez?Db=gene&Cmd=ShowDetailView&TermToSearch=6992) | protein phosphatase 1, regulatory (inhibitor) subunit 11 | -0.74 | 2005, 2007 |
| [PALM2](http://www.ncbi.nlm.nih.gov/sites/entrez?Db=gene&Cmd=ShowDetailView&TermToSearch=114299) | paralemmin 2 | -0.73 |  |
| [FAM76A](http://www.ncbi.nlm.nih.gov/sites/entrez?Db=gene&Cmd=ShowDetailView&TermToSearch=199870) | family with sequence similarity 76, member A | -0.73 | 2007 |
| [MPP2](http://www.ncbi.nlm.nih.gov/sites/entrez?Db=gene&Cmd=ShowDetailView&TermToSearch=4355) | membrane protein, palmitoylated 2 (MAGUK p55 subfamily member 2) | -0.72 | 2005, 2007 |
| [FAM83H](http://www.ncbi.nlm.nih.gov/sites/entrez?Db=gene&Cmd=ShowDetailView&TermToSearch=286077) | family with sequence similarity 83, member H | -0.71 |  |
| [NAV3](http://www.ncbi.nlm.nih.gov/sites/entrez?Db=gene&Cmd=ShowDetailView&TermToSearch=89795) | neuron navigator 3 | -0.7 | 2005, 2007 |
| [MET](http://www.ncbi.nlm.nih.gov/sites/entrez?Db=gene&Cmd=ShowDetailView&TermToSearch=4233) | met proto-oncogene (hepatocyte growth factor receptor) | -0.67 | 2005, 2007 |
| [ZIC5](http://www.ncbi.nlm.nih.gov/sites/entrez?Db=gene&Cmd=ShowDetailView&TermToSearch=85416) | Zic family member 5 (odd-paired homolog, Drosophila) | -0.65 | 2007 |
| [DDX17](http://www.ncbi.nlm.nih.gov/sites/entrez?Db=gene&Cmd=ShowDetailView&TermToSearch=10521) | DEAD (Asp-Glu-Ala-Asp) box polypeptide 17 | -0.64 | 2005, 2007 |
| [CCNE2](http://www.ncbi.nlm.nih.gov/sites/entrez?Db=gene&Cmd=ShowDetailView&TermToSearch=9134) | cyclin E2 | -0.64 | 2005, 2007 |
| [ASB1](http://www.ncbi.nlm.nih.gov/sites/entrez?Db=gene&Cmd=ShowDetailView&TermToSearch=51665) | ankyrin repeat and SOCS box-containing 1 | -0.63 | 2005, 2007 |
| [USP31](http://www.ncbi.nlm.nih.gov/sites/entrez?Db=gene&Cmd=ShowDetailView&TermToSearch=57478) | ubiquitin specific peptidase 31 | -0.63 | 2007 |
| [FAM167A](http://www.ncbi.nlm.nih.gov/sites/entrez?Db=gene&Cmd=ShowDetailView&TermToSearch=83648) | family with sequence similarity 167, member A | -0.62 | 2005, 2007 |
| [PACS1](http://www.ncbi.nlm.nih.gov/sites/entrez?Db=gene&Cmd=ShowDetailView&TermToSearch=55690) | phosphofurin acidic cluster sorting protein 1 | -0.62 | 2005, 2007 |
| [ARHGAP26](http://www.ncbi.nlm.nih.gov/sites/entrez?Db=gene&Cmd=ShowDetailView&TermToSearch=23092) | Rho GTPase activating protein 26 | -0.62 | 2005, 2007 |
| [ING5](http://www.ncbi.nlm.nih.gov/sites/entrez?Db=gene&Cmd=ShowDetailView&TermToSearch=84289) | inhibitor of growth family, member 5 | -0.62 |  |
| [FAM107A](http://www.ncbi.nlm.nih.gov/sites/entrez?Db=gene&Cmd=ShowDetailView&TermToSearch=11170) | family with sequence similarity 107, member A | -0.61 |  |
| [NOTCH1](http://www.ncbi.nlm.nih.gov/sites/entrez?Db=gene&Cmd=ShowDetailView&TermToSearch=4851) | Notch homolog 1, translocation-associated (Drosophila) | -0.6 | 2003, 2005, 2007 |
| [C8orf58](http://www.ncbi.nlm.nih.gov/sites/entrez?Db=gene&Cmd=ShowDetailView&TermToSearch=541565) | chromosome 8 open reading frame 58 | -0.58 |  |
| [PDGFRA](http://www.ncbi.nlm.nih.gov/sites/entrez?Db=gene&Cmd=ShowDetailView&TermToSearch=5156) | platelet-derived growth factor receptor, alpha polypeptide | -0.58 | 2005, 2007 |
| [CNTN2](http://www.ncbi.nlm.nih.gov/sites/entrez?Db=gene&Cmd=ShowDetailView&TermToSearch=6900) | contactin 2 (axonal) | -0.58 | 2005, 2007 |
| [ARHGAP1](http://www.ncbi.nlm.nih.gov/sites/entrez?Db=gene&Cmd=ShowDetailView&TermToSearch=392) | Rho GTPase activating protein 1 | -0.56 | 2005, 2007 |
| [VAMP2](http://www.ncbi.nlm.nih.gov/sites/entrez?Db=gene&Cmd=ShowDetailView&TermToSearch=6844) | vesicle-associated membrane protein 2 (synaptobrevin 2) | -0.56 | 2003, 2005, 2007 |
| [SCN2B](http://www.ncbi.nlm.nih.gov/sites/entrez?Db=gene&Cmd=ShowDetailView&TermToSearch=6327) | sodium channel, voltage-gated, type II, beta | -0.55 | 2005, 2007 |
| [NAV1](http://www.ncbi.nlm.nih.gov/sites/entrez?Db=gene&Cmd=ShowDetailView&TermToSearch=89796) | neuron navigator 1 | -0.55 | 2005, 2007 |
| [TMEM22](http://www.ncbi.nlm.nih.gov/sites/entrez?Db=gene&Cmd=ShowDetailView&TermToSearch=80723) | transmembrane protein 22 | -0.53 |  |
| [SYT1](http://www.ncbi.nlm.nih.gov/sites/entrez?Db=gene&Cmd=ShowDetailView&TermToSearch=6857) | synaptotagmin I | -0.52 | 2005, 2007 |
| [NUMBL](http://www.ncbi.nlm.nih.gov/sites/entrez?Db=gene&Cmd=ShowDetailView&TermToSearch=9253) | numb homolog (Drosophila)-like | -0.51 | 2003, 2005, 2007 |
| [DPYSL4](http://www.ncbi.nlm.nih.gov/sites/entrez?Db=gene&Cmd=ShowDetailView&TermToSearch=10570) | dihydropyrimidinase-like 4 | -0.5 | 2005, 2007 |
| [EI24](http://www.ncbi.nlm.nih.gov/sites/entrez?Db=gene&Cmd=ShowDetailView&TermToSearch=9538) | etoposide induced 2.4 mRNA | -0.5 | 2005, 2007 |
| [ZNF281](http://www.ncbi.nlm.nih.gov/sites/entrez?Db=gene&Cmd=ShowDetailView&TermToSearch=23528) | zinc finger protein 281 | -0.5 | 2005, 2007 |
| [FUT9](http://www.ncbi.nlm.nih.gov/sites/entrez?Db=gene&Cmd=ShowDetailView&TermToSearch=10690) | fucosyltransferase 9 (alpha (1,3) fucosyltransferase) | -0.5 | 2007 |
| [CORO1C](http://www.ncbi.nlm.nih.gov/sites/entrez?Db=gene&Cmd=ShowDetailView&TermToSearch=23603) | coronin, actin binding protein, 1C | -0.49 | 2005, 2007 |
| [**E2F5**](http://www.ncbi.nlm.nih.gov/sites/entrez?Db=gene&Cmd=ShowDetailView&TermToSearch=1875) | **E2F transcription factor 5, p130-binding** | **-0.49** |  |
| [FAM70A](http://www.ncbi.nlm.nih.gov/sites/entrez?Db=gene&Cmd=ShowDetailView&TermToSearch=55026) | family with sequence similarity 70, member A | -0.48 | 2007 |
| [FGD6](http://www.ncbi.nlm.nih.gov/sites/entrez?Db=gene&Cmd=ShowDetailView&TermToSearch=55785) | FYVE, RhoGEF and PH domain containing 6 | -0.48 | 2005, 2007 |
| [GOLPH3L](http://www.ncbi.nlm.nih.gov/sites/entrez?Db=gene&Cmd=ShowDetailView&TermToSearch=55204) | golgi phosphoprotein 3-like | -0.47 |  |
| [SLC44A2](http://www.ncbi.nlm.nih.gov/sites/entrez?Db=gene&Cmd=ShowDetailView&TermToSearch=57153) | solute carrier family 44, member 2 | -0.47 | 2007 |
| [MSL2L1](http://www.ncbi.nlm.nih.gov/sites/entrez?Db=gene&Cmd=ShowDetailView&TermToSearch=55167) | male-specific lethal 2-like 1 (Drosophila) | -0.46 | 2007 |
| [KITLG](http://www.ncbi.nlm.nih.gov/sites/entrez?Db=gene&Cmd=ShowDetailView&TermToSearch=4254) | KIT ligand | -0.46 | 2005, 2007 |
| [PKP4](http://www.ncbi.nlm.nih.gov/sites/entrez?Db=gene&Cmd=ShowDetailView&TermToSearch=8502) | plakophilin 4 | -0.46 | 2005, 2007 |
| [MAP1A](http://www.ncbi.nlm.nih.gov/sites/entrez?Db=gene&Cmd=ShowDetailView&TermToSearch=4130) | microtubule-associated protein 1A | -0.46 | 2005, 2007 |
| [MTA2](http://www.ncbi.nlm.nih.gov/sites/entrez?Db=gene&Cmd=ShowDetailView&TermToSearch=9219) | metastasis associated 1 family, member 2 | -0.45 | 2005, 2007 |
| [FUT8](http://www.ncbi.nlm.nih.gov/sites/entrez?Db=gene&Cmd=ShowDetailView&TermToSearch=2530) | fucosyltransferase 8 (alpha (1,6) fucosyltransferase) | -0.45 | 2005, 2007 |
| [FOXN3](http://www.ncbi.nlm.nih.gov/sites/entrez?Db=gene&Cmd=ShowDetailView&TermToSearch=1112) | forkhead box N3 | -0.45 |  |
| [DBC1](http://www.ncbi.nlm.nih.gov/sites/entrez?Db=gene&Cmd=ShowDetailView&TermToSearch=1620) | deleted in bladder cancer 1 | -0.45 | 2005, 2007 |
| [TAF5](http://www.ncbi.nlm.nih.gov/sites/entrez?Db=gene&Cmd=ShowDetailView&TermToSearch=6877) | TAF5 RNA polymerase II, TATA box binding protein (TBP)-associated factor, 100kDa | -0.44 | 2005, 2007 |
| [FOXP1](http://www.ncbi.nlm.nih.gov/sites/entrez?Db=gene&Cmd=ShowDetailView&TermToSearch=27086) | forkhead box P1 | -0.44 | 2005, 2007 |
| [RRAS](http://www.ncbi.nlm.nih.gov/sites/entrez?Db=gene&Cmd=ShowDetailView&TermToSearch=6237) | related RAS viral (r-ras) oncogene homolog | -0.44 | 2005, 2007 |
| [BMP3](http://www.ncbi.nlm.nih.gov/sites/entrez?Db=gene&Cmd=ShowDetailView&TermToSearch=651) | bone morphogenetic protein 3 | -0.43 |  |
| [ACSL4](http://www.ncbi.nlm.nih.gov/sites/entrez?Db=gene&Cmd=ShowDetailView&TermToSearch=2182) | acyl-CoA synthetase long-chain family member 4 | -0.43 | 2003, 2005, 2007 |
| [FOXP2](http://www.ncbi.nlm.nih.gov/sites/entrez?Db=gene&Cmd=ShowDetailView&TermToSearch=93986) | forkhead box P2 | -0.43 |  |
| [NRIP3](http://www.ncbi.nlm.nih.gov/sites/entrez?Db=gene&Cmd=ShowDetailView&TermToSearch=56675) | nuclear receptor interacting protein 3 | -0.43 | 2005, 2007 |
| [OSGIN2](http://www.ncbi.nlm.nih.gov/sites/entrez?Db=gene&Cmd=ShowDetailView&TermToSearch=734) | oxidative stress induced growth inhibitor family member 2 | -0.42 | 2007 |
| [CBFA2T3](http://www.ncbi.nlm.nih.gov/sites/entrez?Db=gene&Cmd=ShowDetailView&TermToSearch=863) | core-binding factor, runt domain, alpha subunit 2; translocated to, 3 | -0.42 | 2005, 2007 |
| [PIP5K1A](http://www.ncbi.nlm.nih.gov/sites/entrez?Db=gene&Cmd=ShowDetailView&TermToSearch=8394) | phosphatidylinositol-4-phosphate 5-kinase, type I, alpha | -0.42 | 2005, 2007 |
| [SAR1A](http://www.ncbi.nlm.nih.gov/sites/entrez?Db=gene&Cmd=ShowDetailView&TermToSearch=56681) | SAR1 gene homolog A (S. cerevisiae) | -0.42 | 2007 |
| [E2F3](http://www.ncbi.nlm.nih.gov/sites/entrez?Db=gene&Cmd=ShowDetailView&TermToSearch=1871) | E2F transcription factor 3 | -0.42 | 2005, 2007 |
| [KCNE1L](http://www.ncbi.nlm.nih.gov/sites/entrez?Db=gene&Cmd=ShowDetailView&TermToSearch=23630) | KCNE1-like | -0.42 |  |
| [BTBD11](http://www.ncbi.nlm.nih.gov/sites/entrez?Db=gene&Cmd=ShowDetailView&TermToSearch=121551) | BTB (POZ) domain containing 11 | -0.41 | 2005, 2007 |
| [LYST](http://www.ncbi.nlm.nih.gov/sites/entrez?Db=gene&Cmd=ShowDetailView&TermToSearch=1130) | lysosomal trafficking regulator | -0.41 | 2005, 2007 |
| [ITK](http://www.ncbi.nlm.nih.gov/sites/entrez?Db=gene&Cmd=ShowDetailView&TermToSearch=3702) | IL2-inducible T-cell kinase | -0.41 |  |
| [MYCN](http://www.ncbi.nlm.nih.gov/sites/entrez?Db=gene&Cmd=ShowDetailView&TermToSearch=4613) | v-myc myelocytomatosis viral related oncogene, neuroblastoma derived (avian) | -0.4 |  |
| [STRN3](http://www.ncbi.nlm.nih.gov/sites/entrez?Db=gene&Cmd=ShowDetailView&TermToSearch=29966) | striatin, calmodulin binding protein 3 | -0.4 |  |
| [RAB43](http://www.ncbi.nlm.nih.gov/sites/entrez?Db=gene&Cmd=ShowDetailView&TermToSearch=339122) | RAB43, member RAS oncogene family | -0.4 | 2005, 2007 |
| [EVI5L](http://www.ncbi.nlm.nih.gov/sites/entrez?Db=gene&Cmd=ShowDetailView&TermToSearch=115704) | ecotropic viral integration site 5-like | -0.4 | 2005, 2007 |
| [KIAA1045](http://www.ncbi.nlm.nih.gov/sites/entrez?Db=gene&Cmd=ShowDetailView&TermToSearch=23349) | KIAA1045 | -0.4 |  |
| [C5orf43](http://www.ncbi.nlm.nih.gov/sites/entrez?Db=gene&Cmd=ShowDetailView&TermToSearch=643155) | chromosome 5 open reading frame 43 | -0.4 | 2007 |
| [LETMD1](http://www.ncbi.nlm.nih.gov/sites/entrez?Db=gene&Cmd=ShowDetailView&TermToSearch=25875) | LETM1 domain containing 1 | -0.4 |  |
| [TRIM41](http://www.ncbi.nlm.nih.gov/sites/entrez?Db=gene&Cmd=ShowDetailView&TermToSearch=90933) | tripartite motif-containing 41 | -0.4 |  |
| [ZDHHC17](http://www.ncbi.nlm.nih.gov/sites/entrez?Db=gene&Cmd=ShowDetailView&TermToSearch=23390) | zinc finger, DHHC-type containing 17 | -0.4 | 2005, 2007 |
| [POGZ](http://www.ncbi.nlm.nih.gov/sites/entrez?Db=gene&Cmd=ShowDetailView&TermToSearch=23126) | pogo transposable element with ZNF domain | -0.39 | 2005, 2007 |
| [SLC25A27](http://www.ncbi.nlm.nih.gov/sites/entrez?Db=gene&Cmd=ShowDetailView&TermToSearch=9481) | solute carrier family 25, member 27 | -0.38 | 2007 |
| [SIDT1](http://www.ncbi.nlm.nih.gov/sites/entrez?Db=gene&Cmd=ShowDetailView&TermToSearch=54847) | SID1 transmembrane family, member 1 | -0.38 | 2005, 2007 |
| [TMEM97](http://www.ncbi.nlm.nih.gov/sites/entrez?Db=gene&Cmd=ShowDetailView&TermToSearch=27346) | transmembrane protein 97 | -0.38 |  |
| [SCML2](http://www.ncbi.nlm.nih.gov/sites/entrez?Db=gene&Cmd=ShowDetailView&TermToSearch=10389) | sex comb on midleg-like 2 (Drosophila) | -0.38 | 2005, 2007 |
| [EML5](http://www.ncbi.nlm.nih.gov/sites/entrez?Db=gene&Cmd=ShowDetailView&TermToSearch=161436) | echinoderm microtubule associated protein like 5 | -0.38 | 2005, 2007 |
| [CNTNAP2](http://www.ncbi.nlm.nih.gov/sites/entrez?Db=gene&Cmd=ShowDetailView&TermToSearch=26047) | contactin associated protein-like 2 | -0.38 | 2005, 2007 |
| [GPR64](http://www.ncbi.nlm.nih.gov/sites/entrez?Db=gene&Cmd=ShowDetailView&TermToSearch=10149) | G protein-coupled receptor 64 | -0.38 | 2005, 2007 |
| [IL6R](http://www.ncbi.nlm.nih.gov/sites/entrez?Db=gene&Cmd=ShowDetailView&TermToSearch=3570) | interleukin 6 receptor | -0.38 |  |
| [ZNF644](http://www.ncbi.nlm.nih.gov/sites/entrez?Db=gene&Cmd=ShowDetailView&TermToSearch=84146) | zinc finger protein 644 | -0.38 | 2005, 2007 |
| [AP1S2](http://www.ncbi.nlm.nih.gov/sites/entrez?Db=gene&Cmd=ShowDetailView&TermToSearch=8905) | adaptor-related protein complex 1, sigma 2 subunit | -0.38 |  |
| [PVRL1](http://www.ncbi.nlm.nih.gov/sites/entrez?Db=gene&Cmd=ShowDetailView&TermToSearch=5818) | poliovirus receptor-related 1 (herpesvirus entry mediator C) | -0.38 | 2007 |
| [RNF41](http://www.ncbi.nlm.nih.gov/sites/entrez?Db=gene&Cmd=ShowDetailView&TermToSearch=10193) | ring finger protein 41 | -0.37 | 2005, 2007 |
| [5-Mar](http://www.ncbi.nlm.nih.gov/sites/entrez?Db=gene&Cmd=ShowDetailView&TermToSearch=54708) | membrane-associated ring finger (C3HC4) 5 | -0.37 | 2007 |
| [ZMYM4](http://www.ncbi.nlm.nih.gov/sites/entrez?Db=gene&Cmd=ShowDetailView&TermToSearch=9202) | zinc finger, MYM-type 4 | -0.37 | 2007 |
| [MAP2K1](http://www.ncbi.nlm.nih.gov/sites/entrez?Db=gene&Cmd=ShowDetailView&TermToSearch=5604) | mitogen-activated protein kinase kinase 1 | -0.37 | 2007 |
| [AREG](http://www.ncbi.nlm.nih.gov/sites/entrez?Db=gene&Cmd=ShowDetailView&TermToSearch=374) | amphiregulin (schwannoma-derived growth factor) | -0.37 |  |
| [ACCN1](http://www.ncbi.nlm.nih.gov/sites/entrez?Db=gene&Cmd=ShowDetailView&TermToSearch=40) | amiloride-sensitive cation channel 1, neuronal (degenerin) | -0.37 | 2005, 2007 |
| [CALCR](http://www.ncbi.nlm.nih.gov/sites/entrez?Db=gene&Cmd=ShowDetailView&TermToSearch=799) | calcitonin receptor | -0.37 | 2005, 2007 |
| [ADO](http://www.ncbi.nlm.nih.gov/sites/entrez?Db=gene&Cmd=ShowDetailView&TermToSearch=84890) | 2-aminoethanethiol (cysteamine) dioxygenase | -0.36 |  |
| [COL12A1](http://www.ncbi.nlm.nih.gov/sites/entrez?Db=gene&Cmd=ShowDetailView&TermToSearch=1303) | collagen, type XII, alpha 1 | -0.36 | 2005, 2007 |
| [TMEM109](http://www.ncbi.nlm.nih.gov/sites/entrez?Db=gene&Cmd=ShowDetailView&TermToSearch=79073) | transmembrane protein 109 | -0.36 | 2007 |
| [EIF2C4](http://www.ncbi.nlm.nih.gov/sites/entrez?Db=gene&Cmd=ShowDetailView&TermToSearch=192670) | eukaryotic translation initiation factor 2C, 4 | -0.36 | 2005, 2007 |
| [LDHA](http://www.ncbi.nlm.nih.gov/sites/entrez?Db=gene&Cmd=ShowDetailView&TermToSearch=3939) | lactate dehydrogenase A | -0.36 | 2005, 2007 |
| [NONO](http://www.ncbi.nlm.nih.gov/sites/entrez?Db=gene&Cmd=ShowDetailView&TermToSearch=4841) | non-POU domain containing, octamer-binding | -0.36 | 2005, 2007 |
| [MEX3C](http://www.ncbi.nlm.nih.gov/sites/entrez?Db=gene&Cmd=ShowDetailView&TermToSearch=51320) | mex-3 homolog C (C. elegans) | -0.36 | 2005, 2007 |
| [DNAJC16](http://www.ncbi.nlm.nih.gov/sites/entrez?Db=gene&Cmd=ShowDetailView&TermToSearch=23341) | DnaJ (Hsp40) homolog, subfamily C, member 16 | -0.36 | 2007 |
| [DGKZ](http://www.ncbi.nlm.nih.gov/sites/entrez?Db=gene&Cmd=ShowDetailView&TermToSearch=8525) | diacylglycerol kinase, zeta 104kDa | -0.36 | 2005, 2007 |
| [CASP2](http://www.ncbi.nlm.nih.gov/sites/entrez?Db=gene&Cmd=ShowDetailView&TermToSearch=835) | caspase 2, apoptosis-related cysteine peptidase (neural precursor cell expressed, developmentally down-regulated 2) | -0.35 |  |
| [PURB](http://www.ncbi.nlm.nih.gov/sites/entrez?Db=gene&Cmd=ShowDetailView&TermToSearch=5814) | purine-rich element binding protein B | -0.35 | 2005, 2007 |
| [PNOC](http://www.ncbi.nlm.nih.gov/sites/entrez?Db=gene&Cmd=ShowDetailView&TermToSearch=5368) | prepronociceptin | -0.35 | 2005, 2007 |
| [RAP1GDS1](http://www.ncbi.nlm.nih.gov/sites/entrez?Db=gene&Cmd=ShowDetailView&TermToSearch=5910) | RAP1, GTP-GDP dissociation stimulator 1 | -0.35 |  |
| [ProSAPiP1](http://www.ncbi.nlm.nih.gov/sites/entrez?Db=gene&Cmd=ShowDetailView&TermToSearch=9762) | ProSAPiP1 protein | -0.34 | 2005, 2007 |
| [MTUS1](http://www.ncbi.nlm.nih.gov/sites/entrez?Db=gene&Cmd=ShowDetailView&TermToSearch=57509) | mitochondrial tumor suppressor 1 | -0.34 |  |
| [FLOT2](http://www.ncbi.nlm.nih.gov/sites/entrez?Db=gene&Cmd=ShowDetailView&TermToSearch=2319) | flotillin 2 | -0.34 |  |
| [UBP1](http://www.ncbi.nlm.nih.gov/sites/entrez?Db=gene&Cmd=ShowDetailView&TermToSearch=7342) | upstream binding protein 1 (LBP-1a) | -0.34 | 2005, 2007 |
| [PPP2R3A](http://www.ncbi.nlm.nih.gov/sites/entrez?Db=gene&Cmd=ShowDetailView&TermToSearch=5523) | protein phosphatase 2 (formerly 2A), regulatory subunit B', alpha | -0.34 | 2005, 2007 |
| [PLCG1](http://www.ncbi.nlm.nih.gov/sites/entrez?Db=gene&Cmd=ShowDetailView&TermToSearch=5335) | phospholipase C, gamma 1 | -0.34 | 2005, 2007 |
| [PTPRD](http://www.ncbi.nlm.nih.gov/sites/entrez?Db=gene&Cmd=ShowDetailView&TermToSearch=5789) | protein tyrosine phosphatase, receptor type, D | -0.34 |  |
| [SHANK3](http://www.ncbi.nlm.nih.gov/sites/entrez?Db=gene&Cmd=ShowDetailView&TermToSearch=85358) | SH3 and multiple ankyrin repeat domains 3 | -0.34 | 2007 |
| [TPD52](http://www.ncbi.nlm.nih.gov/sites/entrez?Db=gene&Cmd=ShowDetailView&TermToSearch=7163) | tumor protein D52 | -0.34 | 2005, 2007 |
| [FOXJ2](http://www.ncbi.nlm.nih.gov/sites/entrez?Db=gene&Cmd=ShowDetailView&TermToSearch=55810) | forkhead box J2 | -0.33 | 2005, 2007 |
| [THUMPD1](http://www.ncbi.nlm.nih.gov/sites/entrez?Db=gene&Cmd=ShowDetailView&TermToSearch=55623) | THUMP domain containing 1 | -0.33 |  |
| [SLC2A4RG](http://www.ncbi.nlm.nih.gov/sites/entrez?Db=gene&Cmd=ShowDetailView&TermToSearch=56731) | SLC2A4 regulator | -0.33 | 2005, 2007 |
| [PHF15](http://www.ncbi.nlm.nih.gov/sites/entrez?Db=gene&Cmd=ShowDetailView&TermToSearch=23338) | PHD finger protein 15 | -0.33 | 2005, 2007 |
| [NSMCE4A](http://www.ncbi.nlm.nih.gov/sites/entrez?Db=gene&Cmd=ShowDetailView&TermToSearch=54780) | non-SMC element 4 homolog A (S. cerevisiae) | -0.32 |  |
| [JAKMIP1](http://www.ncbi.nlm.nih.gov/sites/entrez?Db=gene&Cmd=ShowDetailView&TermToSearch=152789) | janus kinase and microtubule interacting protein 1 | -0.32 | 2007 |
| [XPO5](http://www.ncbi.nlm.nih.gov/sites/entrez?Db=gene&Cmd=ShowDetailView&TermToSearch=57510) | exportin 5 | -0.32 | 2005, 2007 |
| [GMFB](http://www.ncbi.nlm.nih.gov/sites/entrez?Db=gene&Cmd=ShowDetailView&TermToSearch=2764) | glia maturation factor, beta | -0.32 | 2005, 2007 |
| [FAM123B](http://www.ncbi.nlm.nih.gov/sites/entrez?Db=gene&Cmd=ShowDetailView&TermToSearch=139285) | family with sequence similarity 123B | -0.32 | 2007 |
| [TBL1XR1](http://www.ncbi.nlm.nih.gov/sites/entrez?Db=gene&Cmd=ShowDetailView&TermToSearch=79718) | transducin (beta)-like 1 X-linked receptor 1 | -0.32 | 2005, 2007 |
| [VAT1](http://www.ncbi.nlm.nih.gov/sites/entrez?Db=gene&Cmd=ShowDetailView&TermToSearch=10493) | vesicle amine transport protein 1 homolog (T. californica) | -0.32 | 2005, 2007 |
| [GRM7](http://www.ncbi.nlm.nih.gov/sites/entrez?Db=gene&Cmd=ShowDetailView&TermToSearch=2917) | glutamate receptor, metabotropic 7 | -0.32 | 2007 |
| [NAMPT](http://www.ncbi.nlm.nih.gov/sites/entrez?Db=gene&Cmd=ShowDetailView&TermToSearch=10135) | nicotinamide phosphoribosyltransferase | -0.31 |  |
| [SLC2A13](http://www.ncbi.nlm.nih.gov/sites/entrez?Db=gene&Cmd=ShowDetailView&TermToSearch=114134) | solute carrier family 2 (facilitated glucose transporter), member 13 | -0.31 | 2005, 2007 |
| [CAMTA1](http://www.ncbi.nlm.nih.gov/sites/entrez?Db=gene&Cmd=ShowDetailView&TermToSearch=23261) | calmodulin binding transcription activator 1 | -0.31 | 2005, 2007 |
| [ZDHHC23](http://www.ncbi.nlm.nih.gov/sites/entrez?Db=gene&Cmd=ShowDetailView&TermToSearch=254887) | zinc finger, DHHC-type containing 23 | -0.31 | 2005, 2007 |
| [CSF1R](http://www.ncbi.nlm.nih.gov/sites/entrez?Db=gene&Cmd=ShowDetailView&TermToSearch=1436) | colony stimulating factor 1 receptor, formerly McDonough feline sarcoma viral (v-fms) oncogene homolog | -0.31 | 2005, 2007 |
| [CA7](http://www.ncbi.nlm.nih.gov/sites/entrez?Db=gene&Cmd=ShowDetailView&TermToSearch=766) | carbonic anhydrase VII | -0.31 | 2005, 2007 |
| [ALDOA](http://www.ncbi.nlm.nih.gov/sites/entrez?Db=gene&Cmd=ShowDetailView&TermToSearch=226) | aldolase A, fructose-bisphosphate | -0.31 | 2005, 2007 |
| [MLLT3](http://www.ncbi.nlm.nih.gov/sites/entrez?Db=gene&Cmd=ShowDetailView&TermToSearch=4300) | myeloid/lymphoid or mixed-lineage leukemia (trithorax homolog, Drosophila); translocated to, 3 | -0.31 | 2005, 2007 |
| [BRPF3](http://www.ncbi.nlm.nih.gov/sites/entrez?Db=gene&Cmd=ShowDetailView&TermToSearch=27154) | bromodomain and PHD finger containing, 3 | -0.31 | 2005, 2007 |
| [NTN1](http://www.ncbi.nlm.nih.gov/sites/entrez?Db=gene&Cmd=ShowDetailView&TermToSearch=9423) | netrin 1 | -0.31 |  |
| [MGAT5B](http://www.ncbi.nlm.nih.gov/sites/entrez?Db=gene&Cmd=ShowDetailView&TermToSearch=146664) | mannosyl (alpha-1,6-)-glycoprotein beta-1,6-N-acetyl-glucosaminyltransferase, isozyme B | -0.3 | 2005, 2007 |
| [ORMDL3](http://www.ncbi.nlm.nih.gov/sites/entrez?Db=gene&Cmd=ShowDetailView&TermToSearch=94103) | ORM1-like 3 (S. cerevisiae) | -0.3 |  |
| [CTNND2](http://www.ncbi.nlm.nih.gov/sites/entrez?Db=gene&Cmd=ShowDetailView&TermToSearch=1501) | catenin (cadherin-associated protein), delta 2 (neural plakophilin-related arm-repeat protein) | -0.3 | 2005, 2007 |
| [ITSN1](http://www.ncbi.nlm.nih.gov/sites/entrez?Db=gene&Cmd=ShowDetailView&TermToSearch=6453) | intersectin 1 (SH3 domain protein) | -0.3 | 2005, 2007 |
| [CNOT6L](http://www.ncbi.nlm.nih.gov/sites/entrez?Db=gene&Cmd=ShowDetailView&TermToSearch=246175) | CCR4-NOT transcription complex, subunit 6-like | -0.3 | 2007 |
| [METAP1](http://www.ncbi.nlm.nih.gov/sites/entrez?Db=gene&Cmd=ShowDetailView&TermToSearch=23173) | methionyl aminopeptidase 1 | -0.3 | 2007 |
| [SLC6A1](http://www.ncbi.nlm.nih.gov/sites/entrez?Db=gene&Cmd=ShowDetailView&TermToSearch=6529) | solute carrier family 6 (neurotransmitter transporter, GABA), member 1 | -0.3 |  |
| [ZFHX4](http://www.ncbi.nlm.nih.gov/sites/entrez?Db=gene&Cmd=ShowDetailView&TermToSearch=79776) | zinc finger homeobox 4 | -0.3 | 2005, 2007 |
| [RALGDS](http://www.ncbi.nlm.nih.gov/sites/entrez?Db=gene&Cmd=ShowDetailView&TermToSearch=5900) | ral guanine nucleotide dissociation stimulator | -0.3 | 2005, 2007 |
| [LEF1](http://www.ncbi.nlm.nih.gov/sites/entrez?Db=gene&Cmd=ShowDetailView&TermToSearch=51176) | lymphoid enhancer-binding factor 1 | -0.29 | 2005, 2007 |
| [BNC2](http://www.ncbi.nlm.nih.gov/sites/entrez?Db=gene&Cmd=ShowDetailView&TermToSearch=54796) | basonuclin 2 | -0.29 | 2005, 2007 |
| [RAI14](http://www.ncbi.nlm.nih.gov/sites/entrez?Db=gene&Cmd=ShowDetailView&TermToSearch=26064) | retinoic acid induced 14 | -0.29 |  |
| [HSPA1B](http://www.ncbi.nlm.nih.gov/sites/entrez?Db=gene&Cmd=ShowDetailView&TermToSearch=3304) | heat shock 70kDa protein 1B | -0.29 |  |
| [FOXN2](http://www.ncbi.nlm.nih.gov/sites/entrez?Db=gene&Cmd=ShowDetailView&TermToSearch=3344) | forkhead box N2 | -0.28 | 2007 |
| [BCL2](http://www.ncbi.nlm.nih.gov/sites/entrez?Db=gene&Cmd=ShowDetailView&TermToSearch=596) | B-cell CLL/lymphoma 2 | -0.29 | 2005, 2007 |
| [UHRF2](http://www.ncbi.nlm.nih.gov/sites/entrez?Db=gene&Cmd=ShowDetailView&TermToSearch=115426) | ubiquitin-like, containing PHD and RING finger domains, 2 | -0.28 | 2005, 2007 |
| [AADACL1](http://www.ncbi.nlm.nih.gov/sites/entrez?Db=gene&Cmd=ShowDetailView&TermToSearch=57552) | arylacetamide deacetylase-like 1 | -0.28 | 2005, 2007 |
| [CACNA1E](http://www.ncbi.nlm.nih.gov/sites/entrez?Db=gene&Cmd=ShowDetailView&TermToSearch=777) | calcium channel, voltage-dependent, R type, alpha 1E subunit | -0.28 |  |
| [NOTCH2](http://www.ncbi.nlm.nih.gov/sites/entrez?Db=gene&Cmd=ShowDetailView&TermToSearch=4853) | Notch homolog 2 (Drosophila) | -0.28 | 2005, 2007 |
| [COPS7B](http://www.ncbi.nlm.nih.gov/sites/entrez?Db=gene&Cmd=ShowDetailView&TermToSearch=64708) | COP9 constitutive photomorphogenic homolog subunit 7B (Arabidopsis) | -0.28 | 2005, 2007 |
| [NR4A2](http://www.ncbi.nlm.nih.gov/sites/entrez?Db=gene&Cmd=ShowDetailView&TermToSearch=4929) | nuclear receptor subfamily 4, group A, member 2 | -0.28 | 2005, 2007 |
| [AKAP6](http://www.ncbi.nlm.nih.gov/sites/entrez?Db=gene&Cmd=ShowDetailView&TermToSearch=9472) | A kinase (PRKA) anchor protein 6 | -0.28 | 2005, 2007 |
| [DAAM1](http://www.ncbi.nlm.nih.gov/sites/entrez?Db=gene&Cmd=ShowDetailView&TermToSearch=23002) | dishevelled associated activator of morphogenesis 1 | -0.28 | 2005, 2007 |
| [MRAS](http://www.ncbi.nlm.nih.gov/sites/entrez?Db=gene&Cmd=ShowDetailView&TermToSearch=22808) | muscle RAS oncogene homolog | -0.28 |  |
| [SMC6](http://www.ncbi.nlm.nih.gov/sites/entrez?Db=gene&Cmd=ShowDetailView&TermToSearch=79677) | structural maintenance of chromosomes 6 | -0.28 |  |
| [MGAT4A](http://www.ncbi.nlm.nih.gov/sites/entrez?Db=gene&Cmd=ShowDetailView&TermToSearch=11320) | mannosyl (alpha-1,3-)-glycoprotein beta-1,4-N-acetylglucosaminyltransferase, isozyme A | -0.27 | 2007 |
| [PTMS](http://www.ncbi.nlm.nih.gov/sites/entrez?Db=gene&Cmd=ShowDetailView&TermToSearch=5763) | parathymosin | -0.27 |  |
| [STAT6](http://www.ncbi.nlm.nih.gov/sites/entrez?Db=gene&Cmd=ShowDetailView&TermToSearch=6778) | signal transducer and activator of transcription 6, interleukin-4 induced | -0.27 |  |
| [LGR4](http://www.ncbi.nlm.nih.gov/sites/entrez?Db=gene&Cmd=ShowDetailView&TermToSearch=55366) | leucine-rich repeat-containing G protein-coupled receptor 4 | -0.27 | 2005, 2007 |
| [WDR37](http://www.ncbi.nlm.nih.gov/sites/entrez?Db=gene&Cmd=ShowDetailView&TermToSearch=22884) | WD repeat domain 37 | -0.27 |  |
| [ZER1](http://www.ncbi.nlm.nih.gov/sites/entrez?Db=gene&Cmd=ShowDetailView&TermToSearch=10444) | zer-1 homolog (C. elegans) | -0.27 | 2007 |
| [EDEM3](http://www.ncbi.nlm.nih.gov/sites/entrez?Db=gene&Cmd=ShowDetailView&TermToSearch=80267) | ER degradation enhancer, mannosidase alpha-like 3 | -0.27 |  |
| [MAP3K14](http://www.ncbi.nlm.nih.gov/sites/entrez?Db=gene&Cmd=ShowDetailView&TermToSearch=9020) | mitogen-activated protein kinase kinase kinase 14 | -0.27 |  |
| [ACBD3](http://www.ncbi.nlm.nih.gov/sites/entrez?Db=gene&Cmd=ShowDetailView&TermToSearch=64746) | acyl-Coenzyme A binding domain containing 3 | -0.27 | 2007 |
| [SRPR](http://www.ncbi.nlm.nih.gov/sites/entrez?Db=gene&Cmd=ShowDetailView&TermToSearch=6734) | signal recognition particle receptor ('docking protein') | -0.26 | 2007 |
| [SYNJ1](http://www.ncbi.nlm.nih.gov/sites/entrez?Db=gene&Cmd=ShowDetailView&TermToSearch=8867) | synaptojanin 1 | -0.26 | 2005, 2007 |
| [VCL](http://www.ncbi.nlm.nih.gov/sites/entrez?Db=gene&Cmd=ShowDetailView&TermToSearch=7414) | vinculin | -0.26 | 2005, 2007 |
| [CLOCK](http://www.ncbi.nlm.nih.gov/sites/entrez?Db=gene&Cmd=ShowDetailView&TermToSearch=9575) | clock homolog (mouse) | -0.26 |  |
| [CPLX2](http://www.ncbi.nlm.nih.gov/sites/entrez?Db=gene&Cmd=ShowDetailView&TermToSearch=10814) | complexin 2 | -0.26 | 2005, 2007 |
| [SYN2](http://www.ncbi.nlm.nih.gov/sites/entrez?Db=gene&Cmd=ShowDetailView&TermToSearch=6854) | synapsin II | -0.26 | 2005, 2007 |
| [DCX](http://www.ncbi.nlm.nih.gov/sites/entrez?Db=gene&Cmd=ShowDetailView&TermToSearch=1641) | doublecortex; lissencephaly, X-linked (doublecortin) | -0.26 | 2005, 2007 |
| [FNDC3B](http://www.ncbi.nlm.nih.gov/sites/entrez?Db=gene&Cmd=ShowDetailView&TermToSearch=64778) | fibronectin type III domain containing 3B | -0.25 | 2005, 2007 |
| [NFE2L1](http://www.ncbi.nlm.nih.gov/sites/entrez?Db=gene&Cmd=ShowDetailView&TermToSearch=4779) | nuclear factor (erythroid-derived 2)-like 1 | -0.25 | 2005, 2007 |
| [USP24](http://www.ncbi.nlm.nih.gov/sites/entrez?Db=gene&Cmd=ShowDetailView&TermToSearch=23358) | ubiquitin specific peptidase 24 | -0.25 |  |
| [GPR158](http://www.ncbi.nlm.nih.gov/sites/entrez?Db=gene&Cmd=ShowDetailView&TermToSearch=57512) | G protein-coupled receptor 158 | -0.25 | 2007 |
| [F2RL2](http://www.ncbi.nlm.nih.gov/sites/entrez?Db=gene&Cmd=ShowDetailView&TermToSearch=2151) | coagulation factor II (thrombin) receptor-like 2 | -0.25 | 2005, 2007 |
| [C14orf43](http://www.ncbi.nlm.nih.gov/sites/entrez?Db=gene&Cmd=ShowDetailView&TermToSearch=91748) | chromosome 14 open reading frame 43 | -0.25 | 2007 |
| [GPR22](http://www.ncbi.nlm.nih.gov/sites/entrez?Db=gene&Cmd=ShowDetailView&TermToSearch=2845) | G protein-coupled receptor 22 | -0.25 | 2007 |
| [C14orf28](http://www.ncbi.nlm.nih.gov/sites/entrez?Db=gene&Cmd=ShowDetailView&TermToSearch=122525) | chromosome 14 open reading frame 28 | -0.25 | 2007 |
| [SERPINF2](http://www.ncbi.nlm.nih.gov/sites/entrez?Db=gene&Cmd=ShowDetailView&TermToSearch=5345) | serpin peptidase inhibitor, clade F (alpha-2 antiplasmin, pigment epithelium derived factor), member 2 | -0.24 |  |
| [PGM1](http://www.ncbi.nlm.nih.gov/sites/entrez?Db=gene&Cmd=ShowDetailView&TermToSearch=5236) | phosphoglucomutase 1 | -0.24 | 2005, 2007 |
| [BCL11B](http://www.ncbi.nlm.nih.gov/sites/entrez?Db=gene&Cmd=ShowDetailView&TermToSearch=64919) | B-cell CLL/lymphoma 11B (zinc finger protein) | -0.24 | 2005, 2007 |
| [SKIP](http://www.ncbi.nlm.nih.gov/sites/entrez?Db=gene&Cmd=ShowDetailView&TermToSearch=51763) | skeletal muscle and kidney enriched inositol phosphatase | -0.24 | 2005, 2007 |
| [NDRG4](http://www.ncbi.nlm.nih.gov/sites/entrez?Db=gene&Cmd=ShowDetailView&TermToSearch=65009) | NDRG family member 4 | -0.24 |  |
| [SNX15](http://www.ncbi.nlm.nih.gov/sites/entrez?Db=gene&Cmd=ShowDetailView&TermToSearch=29907) | sorting nexin 15 | -0.24 | 2005, 2007 |
| [XYLT1](http://www.ncbi.nlm.nih.gov/sites/entrez?Db=gene&Cmd=ShowDetailView&TermToSearch=64131) | xylosyltransferase I | -0.24 | 2005, 2007 |
| [NCDN](http://www.ncbi.nlm.nih.gov/sites/entrez?Db=gene&Cmd=ShowDetailView&TermToSearch=23154) | neurochondrin | -0.24 |  |
| [ZNF641](http://www.ncbi.nlm.nih.gov/sites/entrez?Db=gene&Cmd=ShowDetailView&TermToSearch=121274) | zinc finger protein 641 | -0.24 |  |
| [AXL](http://www.ncbi.nlm.nih.gov/sites/entrez?Db=gene&Cmd=ShowDetailView&TermToSearch=558) | AXL receptor tyrosine kinase | -0.24 | 2005, 2007 |
| [ATG4B](http://www.ncbi.nlm.nih.gov/sites/entrez?Db=gene&Cmd=ShowDetailView&TermToSearch=23192) | ATG4 autophagy related 4 homolog B (S. cerevisiae) | -0.24 | 2007 |
| [LIMD2](http://www.ncbi.nlm.nih.gov/sites/entrez?Db=gene&Cmd=ShowDetailView&TermToSearch=80774) | LIM domain containing 2 | -0.24 | 2007 |
| [RTN4RL1](http://www.ncbi.nlm.nih.gov/sites/entrez?Db=gene&Cmd=ShowDetailView&TermToSearch=146760) | reticulon 4 receptor-like 1 | -0.24 | 2005, 2007 |
| [ELMOD1](http://www.ncbi.nlm.nih.gov/sites/entrez?Db=gene&Cmd=ShowDetailView&TermToSearch=55531) | ELMO/CED-12 domain containing 1 | -0.24 | 2005, 2007 |
| [RGS17](http://www.ncbi.nlm.nih.gov/sites/entrez?Db=gene&Cmd=ShowDetailView&TermToSearch=26575) | regulator of G-protein signaling 17 | -0.24 | 2005, 2007 |
| [GLCE](http://www.ncbi.nlm.nih.gov/sites/entrez?Db=gene&Cmd=ShowDetailView&TermToSearch=26035) | glucuronic acid epimerase | -0.24 | 2005, 2007 |
| [SVOP](http://www.ncbi.nlm.nih.gov/sites/entrez?Db=gene&Cmd=ShowDetailView&TermToSearch=55530) | SV2 related protein homolog (rat) | -0.24 | 2007 |
| [CANT1](http://www.ncbi.nlm.nih.gov/sites/entrez?Db=gene&Cmd=ShowDetailView&TermToSearch=124583) | calcium activated nucleotidase 1 | -0.24 |  |
| [AFF4](http://www.ncbi.nlm.nih.gov/sites/entrez?Db=gene&Cmd=ShowDetailView&TermToSearch=27125) | AF4/FMR2 family, member 4 | -0.24 | 2007 |
| [FOXG1](http://www.ncbi.nlm.nih.gov/sites/entrez?Db=gene&Cmd=ShowDetailView&TermToSearch=2290) | forkhead box G1 | -0.24 | 2005, 2007 |
| [JMJD1C](http://www.ncbi.nlm.nih.gov/sites/entrez?Db=gene&Cmd=ShowDetailView&TermToSearch=221037) | jumonji domain containing 1C | -0.23 | 2005, 2007 |
| [SEMA4C](http://www.ncbi.nlm.nih.gov/sites/entrez?Db=gene&Cmd=ShowDetailView&TermToSearch=54910) | sema domain, immunoglobulin domain (Ig), transmembrane domain (TM) and short cytoplasmic domain, (semaphorin) 4C | -0.23 | 2005, 2007 |
| [FAM46A](http://www.ncbi.nlm.nih.gov/sites/entrez?Db=gene&Cmd=ShowDetailView&TermToSearch=55603) | family with sequence similarity 46, member A | -0.23 | 2007 |
| [MYO1C](http://www.ncbi.nlm.nih.gov/sites/entrez?Db=gene&Cmd=ShowDetailView&TermToSearch=4641) | myosin IC | -0.23 | 2007 |
| [PPP1R16B](http://www.ncbi.nlm.nih.gov/sites/entrez?Db=gene&Cmd=ShowDetailView&TermToSearch=26051) | protein phosphatase 1, regulatory (inhibitor) subunit 16B | -0.23 | 2005, 2007 |
| [PEA15](http://www.ncbi.nlm.nih.gov/sites/entrez?Db=gene&Cmd=ShowDetailView&TermToSearch=8682) | phosphoprotein enriched in astrocytes 15 | -0.23 | 2007 |
| [ZC3H4](http://www.ncbi.nlm.nih.gov/sites/entrez?Db=gene&Cmd=ShowDetailView&TermToSearch=23211) | zinc finger CCCH-type containing 4 | -0.23 | 2007 |
| [DIXDC1](http://www.ncbi.nlm.nih.gov/sites/entrez?Db=gene&Cmd=ShowDetailView&TermToSearch=85458) | DIX domain containing 1 | -0.22 | 2005, 2007 |
| [ONECUT2](http://www.ncbi.nlm.nih.gov/sites/entrez?Db=gene&Cmd=ShowDetailView&TermToSearch=9480) | one cut homeobox 2 | -0.22 | 2007 |
| [SNX30](http://www.ncbi.nlm.nih.gov/sites/entrez?Db=gene&Cmd=ShowDetailView&TermToSearch=401548) | sorting nexin family member 30 | -0.22 |  |
| [GRID1](http://www.ncbi.nlm.nih.gov/sites/entrez?Db=gene&Cmd=ShowDetailView&TermToSearch=2894) | glutamate receptor, ionotropic, delta 1 | -0.22 | 2005, 2007 |
| [TMEM106C](http://www.ncbi.nlm.nih.gov/sites/entrez?Db=gene&Cmd=ShowDetailView&TermToSearch=79022) | transmembrane protein 106C | -0.22 |  |
| [POFUT1](http://www.ncbi.nlm.nih.gov/sites/entrez?Db=gene&Cmd=ShowDetailView&TermToSearch=23509) | protein O-fucosyltransferase 1 | -0.22 | 2005, 2007 |
| [TMCC3](http://www.ncbi.nlm.nih.gov/sites/entrez?Db=gene&Cmd=ShowDetailView&TermToSearch=57458) | transmembrane and coiled-coil domain family 3 | -0.22 | 2005, 2007 |
| [SLC7A2](http://www.ncbi.nlm.nih.gov/sites/entrez?Db=gene&Cmd=ShowDetailView&TermToSearch=6542) | solute carrier family 7 (cationic amino acid transporter, y+ system), member 2 | -0.22 |  |
| [SOX4](http://www.ncbi.nlm.nih.gov/sites/entrez?Db=gene&Cmd=ShowDetailView&TermToSearch=6659) | SRY (sex determining region Y)-box 4 | -0.22 | 2005, 2007 |
| [BAZ2A](http://www.ncbi.nlm.nih.gov/sites/entrez?Db=gene&Cmd=ShowDetailView&TermToSearch=11176) | bromodomain adjacent to zinc finger domain, 2A | -0.22 | 2005, 2007 |
| [FAM63B](http://www.ncbi.nlm.nih.gov/sites/entrez?Db=gene&Cmd=ShowDetailView&TermToSearch=54629) | family with sequence similarity 63, member B | -0.22 |  |
| [ACSL1](http://www.ncbi.nlm.nih.gov/sites/entrez?Db=gene&Cmd=ShowDetailView&TermToSearch=2180) | acyl-CoA synthetase long-chain family member 1 | -0.22 | 2005, 2007 |
| [USF1](http://www.ncbi.nlm.nih.gov/sites/entrez?Db=gene&Cmd=ShowDetailView&TermToSearch=7391) | upstream transcription factor 1 | -0.21 |  |
| [WSCD2](http://www.ncbi.nlm.nih.gov/sites/entrez?Db=gene&Cmd=ShowDetailView&TermToSearch=9671) | WSC domain containing 2 | -0.21 | 2005, 2007 |
| [STAC2](http://www.ncbi.nlm.nih.gov/sites/entrez?Db=gene&Cmd=ShowDetailView&TermToSearch=342667) | SH3 and cysteine rich domain 2 | -0.21 | 2005, 2007 |
| [NAT13](http://www.ncbi.nlm.nih.gov/sites/entrez?Db=gene&Cmd=ShowDetailView&TermToSearch=80218) | N-acetyltransferase 13 | -0.21 | 2007 |
| [TNRC18](http://www.ncbi.nlm.nih.gov/sites/entrez?Db=gene&Cmd=ShowDetailView&TermToSearch=84629) | trinucleotide repeat containing 18 | -0.2 |  |
| [GPR85](http://www.ncbi.nlm.nih.gov/sites/entrez?Db=gene&Cmd=ShowDetailView&TermToSearch=54329) | G protein-coupled receptor 85 | -0.2 | 2005, 2007 |
| [PLOD1](http://www.ncbi.nlm.nih.gov/sites/entrez?Db=gene&Cmd=ShowDetailView&TermToSearch=5351) | procollagen-lysine 1, 2-oxoglutarate 5-dioxygenase 1 | -0.2 | 2005, 2007 |
| [PRCD](http://www.ncbi.nlm.nih.gov/sites/entrez?Db=gene&Cmd=ShowDetailView&TermToSearch=768206) | progressive rod-cone degeneration | -0.2 |  |
| [SATB1](http://www.ncbi.nlm.nih.gov/sites/entrez?Db=gene&Cmd=ShowDetailView&TermToSearch=6304) | SATB homeobox 1 | -0.2 | 2005, 2007 |
| [RTF1](http://www.ncbi.nlm.nih.gov/sites/entrez?Db=gene&Cmd=ShowDetailView&TermToSearch=23168) | Rtf1, Paf1/RNA polymerase II complex component, homolog (S. cerevisiae) | -0.2 | 2007 |
| [AHCYL2](http://www.ncbi.nlm.nih.gov/sites/entrez?Db=gene&Cmd=ShowDetailView&TermToSearch=23382) | S-adenosylhomocysteine hydrolase-like 2 | -0.2 | 2005, 2007 |
| [**TGIF2**](http://www.ncbi.nlm.nih.gov/sites/entrez?Db=gene&Cmd=ShowDetailView&TermToSearch=60436) | **TGFB-induced factor homeobox 2** | **-0.2** | **2005, 2007** |
| [INA](http://www.ncbi.nlm.nih.gov/sites/entrez?Db=gene&Cmd=ShowDetailView&TermToSearch=9118) | internexin neuronal intermediate filament protein, alpha | -0.2 |  |
| [SLC27A4](http://www.ncbi.nlm.nih.gov/sites/entrez?Db=gene&Cmd=ShowDetailView&TermToSearch=10999) | solute carrier family 27 (fatty acid transporter), member 4 | -0.2 | 2005, 2007 |
| [CENTG1](http://www.ncbi.nlm.nih.gov/sites/entrez?Db=gene&Cmd=ShowDetailView&TermToSearch=116986) | centaurin, gamma 1 | -0.2 |  |
| [ITCH](http://www.ncbi.nlm.nih.gov/sites/entrez?Db=gene&Cmd=ShowDetailView&TermToSearch=83737) | itchy E3 ubiquitin protein ligase homolog (mouse) | -0.2 | 2005, 2007 |
| [ADIPOR2](http://www.ncbi.nlm.nih.gov/sites/entrez?Db=gene&Cmd=ShowDetailView&TermToSearch=79602) | adiponectin receptor 2 | -0.19 |  |
| [JAG1](http://www.ncbi.nlm.nih.gov/sites/entrez?Db=gene&Cmd=ShowDetailView&TermToSearch=182) | jagged 1 (Alagille syndrome) | -0.19 | 2005, 2007 |
| [GLRA3](http://www.ncbi.nlm.nih.gov/sites/entrez?Db=gene&Cmd=ShowDetailView&TermToSearch=8001) | glycine receptor, alpha 3 | -0.19 |  |
| [TNRC4](http://www.ncbi.nlm.nih.gov/sites/entrez?Db=gene&Cmd=ShowDetailView&TermToSearch=11189) | trinucleotide repeat containing 4 | -0.19 | 2005, 2007 |
| [SEMA4B](http://www.ncbi.nlm.nih.gov/sites/entrez?Db=gene&Cmd=ShowDetailView&TermToSearch=10509) | sema domain, immunoglobulin domain (Ig), transmembrane domain (TM) and short cytoplasmic domain, (semaphorin) 4B | -0.19 | 2007 |
| [NDST1](http://www.ncbi.nlm.nih.gov/sites/entrez?Db=gene&Cmd=ShowDetailView&TermToSearch=3340) | N-deacetylase/N-sulfotransferase (heparan glucosaminyl) 1 | -0.19 | 2007 |
| [ZCCHC17](http://www.ncbi.nlm.nih.gov/sites/entrez?Db=gene&Cmd=ShowDetailView&TermToSearch=51538) | zinc finger, CCHC domain containing 17 | -0.19 | 2007 |
| [KIAA1217](http://www.ncbi.nlm.nih.gov/sites/entrez?Db=gene&Cmd=ShowDetailView&TermToSearch=56243) | KIAA1217 | -0.19 | 2005, 2007 |
| [HNF4G](http://www.ncbi.nlm.nih.gov/sites/entrez?Db=gene&Cmd=ShowDetailView&TermToSearch=3174) | hepatocyte nuclear factor 4, gamma | -0.19 |  |
| [ANKRD52](http://www.ncbi.nlm.nih.gov/sites/entrez?Db=gene&Cmd=ShowDetailView&TermToSearch=283373) | ankyrin repeat domain 52 | -0.19 |  |
| [FNDC8](http://www.ncbi.nlm.nih.gov/sites/entrez?Db=gene&Cmd=ShowDetailView&TermToSearch=54752) | fibronectin type III domain containing 8 | -0.19 | 2003, 2007 |
| [LGI1](http://www.ncbi.nlm.nih.gov/sites/entrez?Db=gene&Cmd=ShowDetailView&TermToSearch=9211) | leucine-rich, glioma inactivated 1 | -0.19 | 2005, 2007 |
| [PTPRM](http://www.ncbi.nlm.nih.gov/sites/entrez?Db=gene&Cmd=ShowDetailView&TermToSearch=5797) | protein tyrosine phosphatase, receptor type, M | -0.19 | 2005, 2007 |
| [CNTNAP1](http://www.ncbi.nlm.nih.gov/sites/entrez?Db=gene&Cmd=ShowDetailView&TermToSearch=8506) | contactin associated protein 1 | -0.18 | 2005, 2007 |
| [PKIA](http://www.ncbi.nlm.nih.gov/sites/entrez?Db=gene&Cmd=ShowDetailView&TermToSearch=5569) | protein kinase (cAMP-dependent, catalytic) inhibitor alpha | -0.18 | 2005, 2007 |
| [SPCS2](http://www.ncbi.nlm.nih.gov/sites/entrez?Db=gene&Cmd=ShowDetailView&TermToSearch=9789) | signal peptidase complex subunit 2 homolog (S. cerevisiae) | -0.18 | 2005, 2007 |
| [NRXN2](http://www.ncbi.nlm.nih.gov/sites/entrez?Db=gene&Cmd=ShowDetailView&TermToSearch=9379) | neurexin 2 | -0.18 | 2005, 2007 |
| [IGFBP3](http://www.ncbi.nlm.nih.gov/sites/entrez?Db=gene&Cmd=ShowDetailView&TermToSearch=3486) | insulin-like growth factor binding protein 3 | -0.18 | 2005, 2007 |
| [RET](http://www.ncbi.nlm.nih.gov/sites/entrez?Db=gene&Cmd=ShowDetailView&TermToSearch=5979) | ret proto-oncogene | -0.18 |  |
| [FRMD4A](http://www.ncbi.nlm.nih.gov/sites/entrez?Db=gene&Cmd=ShowDetailView&TermToSearch=55691) | FERM domain containing 4A | -0.18 | 2005, 2007 |
| [MRPL52](http://www.ncbi.nlm.nih.gov/sites/entrez?Db=gene&Cmd=ShowDetailView&TermToSearch=122704) | mitochondrial ribosomal protein L52 | -0.18 |  |
| [SGSM2](http://www.ncbi.nlm.nih.gov/sites/entrez?Db=gene&Cmd=ShowDetailView&TermToSearch=9905) | small G protein signaling modulator 2 | -0.18 | 2005, 2007 |
| [SMAD4](http://www.ncbi.nlm.nih.gov/sites/entrez?Db=gene&Cmd=ShowDetailView&TermToSearch=4089) | SMAD family member 4 | -0.18 |  |
| [ASCL1](http://www.ncbi.nlm.nih.gov/sites/entrez?Db=gene&Cmd=ShowDetailView&TermToSearch=429) | achaete-scute complex homolog 1 (Drosophila) | -0.17 |  |
| [YTHDC1](http://www.ncbi.nlm.nih.gov/sites/entrez?Db=gene&Cmd=ShowDetailView&TermToSearch=91746) | YTH domain containing 1 | -0.17 | 2007 |
| [RPS6KA4](http://www.ncbi.nlm.nih.gov/sites/entrez?Db=gene&Cmd=ShowDetailView&TermToSearch=8986) | ribosomal protein S6 kinase, 90kDa, polypeptide 4 | -0.17 | 2005, 2007 |
| [FBXO10](http://www.ncbi.nlm.nih.gov/sites/entrez?Db=gene&Cmd=ShowDetailView&TermToSearch=26267) | F-box protein 10 | -0.17 | 2007 |
| [SLC30A3](http://www.ncbi.nlm.nih.gov/sites/entrez?Db=gene&Cmd=ShowDetailView&TermToSearch=7781) | solute carrier family 30 (zinc transporter), member 3 | -0.17 | 2005, 2007 |
| [GATAD2B](http://www.ncbi.nlm.nih.gov/sites/entrez?Db=gene&Cmd=ShowDetailView&TermToSearch=57459) | GATA zinc finger domain containing 2B | -0.17 |  |
| [KIAA1688](http://www.ncbi.nlm.nih.gov/sites/entrez?Db=gene&Cmd=ShowDetailView&TermToSearch=80728) | KIAA1688 protein | -0.17 |  |
| [PID1](http://www.ncbi.nlm.nih.gov/sites/entrez?Db=gene&Cmd=ShowDetailView&TermToSearch=55022) | phosphotyrosine interaction domain containing 1 | -0.17 | 2007 |
| [DKFZP564O0823](http://www.ncbi.nlm.nih.gov/sites/entrez?Db=gene&Cmd=ShowDetailView&TermToSearch=25849) | DKFZP564O0823 protein | -0.17 |  |
| [CDC25A](http://www.ncbi.nlm.nih.gov/sites/entrez?Db=gene&Cmd=ShowDetailView&TermToSearch=993) | cell division cycle 25 homolog A (S. pombe) | -0.17 | 2005, 2007 |
| [STX17](http://www.ncbi.nlm.nih.gov/sites/entrez?Db=gene&Cmd=ShowDetailView&TermToSearch=55014) | syntaxin 17 | -0.17 | 2005, 2007 |
| [KIAA0100](http://www.ncbi.nlm.nih.gov/sites/entrez?Db=gene&Cmd=ShowDetailView&TermToSearch=9703) | KIAA0100 | -0.17 |  |
| [TOB2](http://www.ncbi.nlm.nih.gov/sites/entrez?Db=gene&Cmd=ShowDetailView&TermToSearch=10766) | transducer of ERBB2, 2 | -0.17 | 2005, 2007 |
| [**INHBB**](http://www.ncbi.nlm.nih.gov/sites/entrez?Db=gene&Cmd=ShowDetailView&TermToSearch=3625) | **inhibin, beta B** | **-0.16** | **2005, 2007** |
| [STX1A](http://www.ncbi.nlm.nih.gov/sites/entrez?Db=gene&Cmd=ShowDetailView&TermToSearch=6804) | syntaxin 1A (brain) | -0.16 |  |
| [CUEDC1](http://www.ncbi.nlm.nih.gov/sites/entrez?Db=gene&Cmd=ShowDetailView&TermToSearch=404093) | CUE domain containing 1 | -0.16 | 2007 |
| [TMEM200B](http://www.ncbi.nlm.nih.gov/sites/entrez?Db=gene&Cmd=ShowDetailView&TermToSearch=399474) | transmembrane protein 200B | -0.16 | 2005, 2007 |
| [C3orf70](http://www.ncbi.nlm.nih.gov/sites/entrez?Db=gene&Cmd=ShowDetailView&TermToSearch=285382) | chromosome 3 open reading frame 70 | -0.16 | 2007 |
| [TANC2](http://www.ncbi.nlm.nih.gov/sites/entrez?Db=gene&Cmd=ShowDetailView&TermToSearch=26115) | tetratricopeptide repeat, ankyrin repeat and coiled-coil containing 2 | -0.16 |  |
| [8-Mar](http://www.ncbi.nlm.nih.gov/sites/entrez?Db=gene&Cmd=ShowDetailView&TermToSearch=220972) | membrane-associated ring finger (C3HC4) 8 | -0.16 |  |
| [EEA1](http://www.ncbi.nlm.nih.gov/sites/entrez?Db=gene&Cmd=ShowDetailView&TermToSearch=8411) | early endosome antigen 1 | -0.16 | 2007 |
| [TCF12](http://www.ncbi.nlm.nih.gov/sites/entrez?Db=gene&Cmd=ShowDetailView&TermToSearch=6938) | transcription factor 12 (HTF4, helix-loop-helix transcription factors 4) | -0.16 | 2005, 2007 |
| [TFRC](http://www.ncbi.nlm.nih.gov/sites/entrez?Db=gene&Cmd=ShowDetailView&TermToSearch=7037) | transferrin receptor (p90, CD71) | -0.16 |  |
| [YY1](http://www.ncbi.nlm.nih.gov/sites/entrez?Db=gene&Cmd=ShowDetailView&TermToSearch=7528) | YY1 transcription factor | -0.16 | 2005, 2007 |
| [RIC8B](http://www.ncbi.nlm.nih.gov/sites/entrez?Db=gene&Cmd=ShowDetailView&TermToSearch=55188) | resistance to inhibitors of cholinesterase 8 homolog B (C. elegans) | -0.15 | 2007 |
| [PRKD1](http://www.ncbi.nlm.nih.gov/sites/entrez?Db=gene&Cmd=ShowDetailView&TermToSearch=5587) | protein kinase D1 | -0.15 | 2005, 2007 |
| [PHF19](http://www.ncbi.nlm.nih.gov/sites/entrez?Db=gene&Cmd=ShowDetailView&TermToSearch=26147) | PHD finger protein 19 | -0.15 | 2005, 2007 |
| [CCND1](http://www.ncbi.nlm.nih.gov/sites/entrez?Db=gene&Cmd=ShowDetailView&TermToSearch=595) | cyclin D1 | -0.15 | 2005, 2007 |
| [CACNB3](http://www.ncbi.nlm.nih.gov/sites/entrez?Db=gene&Cmd=ShowDetailView&TermToSearch=784) | calcium channel, voltage-dependent, beta 3 subunit | -0.15 | 2005, 2007 |
| [DCP1A](http://www.ncbi.nlm.nih.gov/sites/entrez?Db=gene&Cmd=ShowDetailView&TermToSearch=55802) | DCP1 decapping enzyme homolog A (S. cerevisiae) | -0.15 |  |
| [CSNK1G1](http://www.ncbi.nlm.nih.gov/sites/entrez?Db=gene&Cmd=ShowDetailView&TermToSearch=53944) | casein kinase 1, gamma 1 | -0.15 | 2007 |
| [SEC16A](http://www.ncbi.nlm.nih.gov/sites/entrez?Db=gene&Cmd=ShowDetailView&TermToSearch=9919) | SEC16 homolog A (S. cerevisiae) | -0.15 |  |
| [SOX12](http://www.ncbi.nlm.nih.gov/sites/entrez?Db=gene&Cmd=ShowDetailView&TermToSearch=6666) | SRY (sex determining region Y)-box 12 | -0.15 |  |
| [GIGYF1](http://www.ncbi.nlm.nih.gov/sites/entrez?Db=gene&Cmd=ShowDetailView&TermToSearch=64599) | GRB10 interacting GYF protein 1 | -0.14 | 2007 |
| [UBE2G1](http://www.ncbi.nlm.nih.gov/sites/entrez?Db=gene&Cmd=ShowDetailView&TermToSearch=7326) | ubiquitin-conjugating enzyme E2G 1 (UBC7 homolog, yeast) | -0.14 |  |
| [CRHR1](http://www.ncbi.nlm.nih.gov/sites/entrez?Db=gene&Cmd=ShowDetailView&TermToSearch=1394) | corticotropin releasing hormone receptor 1 | -0.14 | 2005, 2007 |
| [PEF1](http://www.ncbi.nlm.nih.gov/sites/entrez?Db=gene&Cmd=ShowDetailView&TermToSearch=553115) | penta-EF-hand domain containing 1 | -0.14 |  |
| [KCNH7](http://www.ncbi.nlm.nih.gov/sites/entrez?Db=gene&Cmd=ShowDetailView&TermToSearch=90134) | potassium voltage-gated channel, subfamily H (eag-related), member 7 | -0.14 | 2007 |
| [CACNB1](http://www.ncbi.nlm.nih.gov/sites/entrez?Db=gene&Cmd=ShowDetailView&TermToSearch=782) | calcium channel, voltage-dependent, beta 1 subunit | -0.14 | 2005, 2007 |
| [CAMSAP1](http://www.ncbi.nlm.nih.gov/sites/entrez?Db=gene&Cmd=ShowDetailView&TermToSearch=157922) | calmodulin regulated spectrin-associated protein 1 | -0.14 | 2005, 2007 |
| [PPFIA1](http://www.ncbi.nlm.nih.gov/sites/entrez?Db=gene&Cmd=ShowDetailView&TermToSearch=8500) | protein tyrosine phosphatase, receptor type, f polypeptide (PTPRF), interacting protein (liprin), alpha 1 | -0.14 | 2005, 2007 |
| [SURF4](http://www.ncbi.nlm.nih.gov/sites/entrez?Db=gene&Cmd=ShowDetailView&TermToSearch=6836) | surfeit 4 | -0.14 |  |
| [TOX](http://www.ncbi.nlm.nih.gov/sites/entrez?Db=gene&Cmd=ShowDetailView&TermToSearch=9760) | thymocyte selection-associated high mobility group box | -0.13 |  |
| [SHKBP1](http://www.ncbi.nlm.nih.gov/sites/entrez?Db=gene&Cmd=ShowDetailView&TermToSearch=92799) | SH3KBP1 binding protein 1 | -0.13 | 2005, 2007 |
| [PGRMC2](http://www.ncbi.nlm.nih.gov/sites/entrez?Db=gene&Cmd=ShowDetailView&TermToSearch=10424) | progesterone receptor membrane component 2 | -0.13 | 2007 |
| [ESRRA](http://www.ncbi.nlm.nih.gov/sites/entrez?Db=gene&Cmd=ShowDetailView&TermToSearch=2101) | estrogen-related receptor alpha | -0.13 | 2005, 2007 |
| [TBC1D25](http://www.ncbi.nlm.nih.gov/sites/entrez?Db=gene&Cmd=ShowDetailView&TermToSearch=4943) | TBC1 domain family, member 25 | -0.13 | 2007 |
| [ARID4A](http://www.ncbi.nlm.nih.gov/sites/entrez?Db=gene&Cmd=ShowDetailView&TermToSearch=5926) | AT rich interactive domain 4A (RBP1-like) | -0.13 | 2005, 2007 |
| [SPRN](http://www.ncbi.nlm.nih.gov/sites/entrez?Db=gene&Cmd=ShowDetailView&TermToSearch=503542) | shadow of prion protein homolog (zebrafish) | -0.12 | 2007 |
| [UBE2L3](http://www.ncbi.nlm.nih.gov/sites/entrez?Db=gene&Cmd=ShowDetailView&TermToSearch=7332) | ubiquitin-conjugating enzyme E2L 3 | -0.12 |  |
| [OXSR1](http://www.ncbi.nlm.nih.gov/sites/entrez?Db=gene&Cmd=ShowDetailView&TermToSearch=9943) | oxidative-stress responsive 1 | -0.12 | 2005, 2007 |
| [SCN1A](http://www.ncbi.nlm.nih.gov/sites/entrez?Db=gene&Cmd=ShowDetailView&TermToSearch=6323) | sodium channel, voltage-gated, type I, alpha subunit | -0.12 | 2007 |
| [SOCS4](http://www.ncbi.nlm.nih.gov/sites/entrez?Db=gene&Cmd=ShowDetailView&TermToSearch=122809) | suppressor of cytokine signaling 4 | -0.12 | 2005, 2007 |
| [tcag7.1228](http://www.ncbi.nlm.nih.gov/sites/entrez?Db=gene&Cmd=ShowDetailView&TermToSearch=254048) | hypothetical protein FLJ25778 | -0.12 |  |
| [SEMA4F](http://www.ncbi.nlm.nih.gov/sites/entrez?Db=gene&Cmd=ShowDetailView&TermToSearch=10505) | sema domain, immunoglobulin domain (Ig), transmembrane domain (TM) and short cytoplasmic domain, (semaphorin) 4F | -0.11 | 2007 |
| [RAPH1](http://www.ncbi.nlm.nih.gov/sites/entrez?Db=gene&Cmd=ShowDetailView&TermToSearch=65059) | Ras association (RalGDS/AF-6) and pleckstrin homology domains 1 | -0.11 |  |
| [TUSC5](http://www.ncbi.nlm.nih.gov/sites/entrez?Db=gene&Cmd=ShowDetailView&TermToSearch=286753) | tumor suppressor candidate 5 | -0.11 |  |
| [3-Sep](http://www.ncbi.nlm.nih.gov/sites/entrez?Db=gene&Cmd=ShowDetailView&TermToSearch=55964) | septin 3 | -0.11 | 2005, 2007 |
| [USP54](http://www.ncbi.nlm.nih.gov/sites/entrez?Db=gene&Cmd=ShowDetailView&TermToSearch=159195) | ubiquitin specific peptidase 54 | -0.11 |  |
| [B4GALT2](http://www.ncbi.nlm.nih.gov/sites/entrez?Db=gene&Cmd=ShowDetailView&TermToSearch=8704) | UDP-Gal:betaGlcNAc beta 1,4- galactosyltransferase, polypeptide 2 | -0.11 | 2007 |
| [SLC12A2](http://www.ncbi.nlm.nih.gov/sites/entrez?Db=gene&Cmd=ShowDetailView&TermToSearch=6558) | solute carrier family 12 (sodium/potassium/chloride transporters), member 2 | -0.11 | 2005, 2007 |
| [CACNA2D2](http://www.ncbi.nlm.nih.gov/sites/entrez?Db=gene&Cmd=ShowDetailView&TermToSearch=9254) | calcium channel, voltage-dependent, alpha 2/delta subunit 2 | -0.1 |  |
| [SYVN1](http://www.ncbi.nlm.nih.gov/sites/entrez?Db=gene&Cmd=ShowDetailView&TermToSearch=84447) | synovial apoptosis inhibitor 1, synoviolin | -0.1 | 2005, 2007 |
| [MYRIP](http://www.ncbi.nlm.nih.gov/sites/entrez?Db=gene&Cmd=ShowDetailView&TermToSearch=25924) | myosin VIIA and Rab interacting protein | -0.1 | 2005, 2007 |
| [RDH11](http://www.ncbi.nlm.nih.gov/sites/entrez?Db=gene&Cmd=ShowDetailView&TermToSearch=51109) | retinol dehydrogenase 11 (all-trans/9-cis/11-cis) | -0.1 | 2005, 2007 |
| [CPEB2](http://www.ncbi.nlm.nih.gov/sites/entrez?Db=gene&Cmd=ShowDetailView&TermToSearch=132864) | cytoplasmic polyadenylation element binding protein 2 | -0.1 | 2005, 2007 |
| [TNRC6B](http://www.ncbi.nlm.nih.gov/sites/entrez?Db=gene&Cmd=ShowDetailView&TermToSearch=23112) | trinucleotide repeat containing 6B | -0.1 | 2007 |
| [CDK6](http://www.ncbi.nlm.nih.gov/sites/entrez?Db=gene&Cmd=ShowDetailView&TermToSearch=1021) | cyclin-dependent kinase 6 | -0.1 | 2007 |
| [SLC4A7](http://www.ncbi.nlm.nih.gov/sites/entrez?Db=gene&Cmd=ShowDetailView&TermToSearch=9497) | solute carrier family 4, sodium bicarbonate cotransporter, member 7 | -0.1 | 2005, 2007 |
| [LRRC55](http://www.ncbi.nlm.nih.gov/sites/entrez?Db=gene&Cmd=ShowDetailView&TermToSearch=219527) | leucine rich repeat containing 55 | -0.09 | 2007 |
| [ST8SIA3](http://www.ncbi.nlm.nih.gov/sites/entrez?Db=gene&Cmd=ShowDetailView&TermToSearch=51046) | ST8 alpha-N-acetyl-neuraminide alpha-2,8-sialyltransferase 3 | -0.09 |  |
| [PDCD4](http://www.ncbi.nlm.nih.gov/sites/entrez?Db=gene&Cmd=ShowDetailView&TermToSearch=27250) | programmed cell death 4 (neoplastic transformation inhibitor) | -0.09 |  |
| [PODXL](http://www.ncbi.nlm.nih.gov/sites/entrez?Db=gene&Cmd=ShowDetailView&TermToSearch=5420) | podocalyxin-like | -0.09 | 2005, 2007 |
| [ACTR1A](http://www.ncbi.nlm.nih.gov/sites/entrez?Db=gene&Cmd=ShowDetailView&TermToSearch=10121) | ARP1 actin-related protein 1 homolog A, centractin alpha (yeast) | -0.09 | 2005, 2007 |
| [PLEKHG3](http://www.ncbi.nlm.nih.gov/sites/entrez?Db=gene&Cmd=ShowDetailView&TermToSearch=26030) | pleckstrin homology domain containing, family G (with RhoGef domain) member 3 | -0.09 |  |
| [RNF44](http://www.ncbi.nlm.nih.gov/sites/entrez?Db=gene&Cmd=ShowDetailView&TermToSearch=22838) | ring finger protein 44 | -0.09 | 2005, 2007 |
| [ABR](http://www.ncbi.nlm.nih.gov/sites/entrez?Db=gene&Cmd=ShowDetailView&TermToSearch=29) | active BCR-related gene | -0.09 | 2005, 2007 |
| [ASXL1](http://www.ncbi.nlm.nih.gov/sites/entrez?Db=gene&Cmd=ShowDetailView&TermToSearch=171023) | additional sex combs like 1 (Drosophila) | -0.09 |  |
| [SDHC](http://www.ncbi.nlm.nih.gov/sites/entrez?Db=gene&Cmd=ShowDetailView&TermToSearch=6391) | succinate dehydrogenase complex, subunit C, integral membrane protein, 15kDa | -0.08 | 2005, 2007 |
| [WNT1](http://www.ncbi.nlm.nih.gov/sites/entrez?Db=gene&Cmd=ShowDetailView&TermToSearch=7471) | wingless-type MMTV integration site family, member 1 | -0.08 | 2005, 2007 |
| [ANK3](http://www.ncbi.nlm.nih.gov/sites/entrez?Db=gene&Cmd=ShowDetailView&TermToSearch=288) | ankyrin 3, node of Ranvier (ankyrin G) | -0.08 | 2005, 2007 |
| [FBXO41](http://www.ncbi.nlm.nih.gov/sites/entrez?Db=gene&Cmd=ShowDetailView&TermToSearch=150726) | F-box protein 41 | -0.08 | 2007 |
| [GRHL2](http://www.ncbi.nlm.nih.gov/sites/entrez?Db=gene&Cmd=ShowDetailView&TermToSearch=79977) | grainyhead-like 2 (Drosophila) | -0.08 |  |
| [MLL2](http://www.ncbi.nlm.nih.gov/sites/entrez?Db=gene&Cmd=ShowDetailView&TermToSearch=8085) | myeloid/lymphoid or mixed-lineage leukemia 2 | -0.08 | 2007 |
| [ATG9A](http://www.ncbi.nlm.nih.gov/sites/entrez?Db=gene&Cmd=ShowDetailView&TermToSearch=79065) | ATG9 autophagy related 9 homolog A (S. cerevisiae) | -0.08 | 2007 |
| [ARHGEF3](http://www.ncbi.nlm.nih.gov/sites/entrez?Db=gene&Cmd=ShowDetailView&TermToSearch=50650) | Rho guanine nucleotide exchange factor (GEF) 3 | -0.08 |  |
| [GNAI2](http://www.ncbi.nlm.nih.gov/sites/entrez?Db=gene&Cmd=ShowDetailView&TermToSearch=2771) | guanine nucleotide binding protein (G protein), alpha inhibiting activity polypeptide 2 | -0.07 | 2005, 2007 |
| [KIAA1024](http://www.ncbi.nlm.nih.gov/sites/entrez?Db=gene&Cmd=ShowDetailView&TermToSearch=23251) | KIAA1024 | -0.07 |  |
| [C15orf53](http://www.ncbi.nlm.nih.gov/sites/entrez?Db=gene&Cmd=ShowDetailView&TermToSearch=400359) | chromosome 15 open reading frame 53 | -0.07 | 2005, 2007 |
| [MCFD2](http://www.ncbi.nlm.nih.gov/sites/entrez?Db=gene&Cmd=ShowDetailView&TermToSearch=90411) | multiple coagulation factor deficiency 2 | -0.07 | 2005, 2007 |
| [C5orf41](http://www.ncbi.nlm.nih.gov/sites/entrez?Db=gene&Cmd=ShowDetailView&TermToSearch=153222) | chromosome 5 open reading frame 41 | -0.06 | 2005, 2007 |
| [CNOT6](http://www.ncbi.nlm.nih.gov/sites/entrez?Db=gene&Cmd=ShowDetailView&TermToSearch=57472) | CCR4-NOT transcription complex, subunit 6 | -0.06 | 2005, 2007 |
| [ATP2B4](http://www.ncbi.nlm.nih.gov/sites/entrez?Db=gene&Cmd=ShowDetailView&TermToSearch=493) | ATPase, Ca++ transporting, plasma membrane 4 | -0.05 |  |
| [PTPRE](http://www.ncbi.nlm.nih.gov/sites/entrez?Db=gene&Cmd=ShowDetailView&TermToSearch=5791) | protein tyrosine phosphatase, receptor type, E | -0.05 |  |
| [RALGPS1](http://www.ncbi.nlm.nih.gov/sites/entrez?Db=gene&Cmd=ShowDetailView&TermToSearch=9649) | Ral GEF with PH domain and SH3 binding motif 1 | -0.05 | 2005, 2007 |
| [CTNND1](http://www.ncbi.nlm.nih.gov/sites/entrez?Db=gene&Cmd=ShowDetailView&TermToSearch=1500) | catenin (cadherin-associated protein), delta 1 | -0.05 |  |
| [SERPINE1](http://www.ncbi.nlm.nih.gov/sites/entrez?Db=gene&Cmd=ShowDetailView&TermToSearch=5054) | serpin peptidase inhibitor, clade E (nexin, plasminogen activator inhibitor type 1), member 1 | -0.04 | 2005, 2007 |
| [ORAI3](http://www.ncbi.nlm.nih.gov/sites/entrez?Db=gene&Cmd=ShowDetailView&TermToSearch=93129) | ORAI calcium release-activated calcium modulator 3 | -0.04 |  |
| [RAB11FIP4](http://www.ncbi.nlm.nih.gov/sites/entrez?Db=gene&Cmd=ShowDetailView&TermToSearch=84440) | RAB11 family interacting protein 4 (class II) | -0.04 | 2005, 2007 |
| [TXNDC4](http://www.ncbi.nlm.nih.gov/sites/entrez?Db=gene&Cmd=ShowDetailView&TermToSearch=23071) | thioredoxin domain containing 4 (endoplasmic reticulum) | -0.04 |  |
| [ATXN7L3](http://www.ncbi.nlm.nih.gov/sites/entrez?Db=gene&Cmd=ShowDetailView&TermToSearch=56970) | ataxin 7-like 3 | -0.03 |  |
| [JPH3](http://www.ncbi.nlm.nih.gov/sites/entrez?Db=gene&Cmd=ShowDetailView&TermToSearch=57338) | junctophilin 3 | -0.03 |  |
| [SPRY3](http://www.ncbi.nlm.nih.gov/sites/entrez?Db=gene&Cmd=ShowDetailView&TermToSearch=10251) | sprouty homolog 3 (Drosophila) | -0.02 | 2005, 2007 |
| [LOC285636](http://www.ncbi.nlm.nih.gov/sites/entrez?Db=gene&Cmd=ShowDetailView&TermToSearch=285636) | UPF0600 protein | -0.02 |  |
| [LPHN1](http://www.ncbi.nlm.nih.gov/sites/entrez?Db=gene&Cmd=ShowDetailView&TermToSearch=22859) | latrophilin 1 | -0.01 | 2005, 2007 |
| [ITGA10](http://www.ncbi.nlm.nih.gov/sites/entrez?Db=gene&Cmd=ShowDetailView&TermToSearch=8515) | integrin, alpha 10 | 0 |  |
| [RIMS3](http://www.ncbi.nlm.nih.gov/sites/entrez?Db=gene&Cmd=ShowDetailView&TermToSearch=9783) | regulating synaptic membrane exocytosis 3 | 0.02 | 2005, 2007 |
| [EEF2K](http://www.ncbi.nlm.nih.gov/sites/entrez?Db=gene&Cmd=ShowDetailView&TermToSearch=29904) | eukaryotic elongation factor-2 kinase | N/A | 2007 |
| [CYCS](http://www.ncbi.nlm.nih.gov/sites/entrez?Db=gene&Cmd=ShowDetailView&TermToSearch=54205) | cytochrome c, somatic | N/A |  |
| [CADM4](http://www.ncbi.nlm.nih.gov/sites/entrez?Db=gene&Cmd=ShowDetailView&TermToSearch=199731) | cell adhesion molecule 4 | N/A |  |
