# Supplementary material for: Mir-34a Is Upregulated during Liver Regeneration in Rats and Is Associated with the Suppression of Hepatocyte Proliferation
Source: PLoS One. 2011 May 31;6(5):e20238. doi: 10.1371/journal.pone.0020238 (PMC3105003; doi:10.1371/journal.pone.0020238)
Supplement: Table S2 — Pathways analysis of miR-34a candidate genes using MAS software ( http://bioinfo.capitalbio.com/mas ). Three pathways related to the termination stage of liver regeneration are listed. (DOCX) [file pone.0020238.s002.docx]

**Table S2.**

| **PathwayName** | **Total** | **Pvalue** | **Gene** |
| --- | --- | --- | --- |
| Cell cycle | 6 | 5.01E-4 | SMAD4 |
| Cell cycle | 6 | 5.01E-4 | CDK6 |
| Cell cycle | 6 | 5.01E-4 | CDC25A |
| Cell cycle | 6 | 5.01E-4 | E2F5 |
| Cell cycle | 6 | 5.01E-4 | CCND1 |
| Cell cycle | 6 | 5.01E-4 | CCNE2 |
| TGF-beta signaling pathway | 3 | 0.038735 | SMAD4 |
| TGF-beta signaling pathway | 3 | 0.038735 | E2F5 |
| TGF-beta signaling pathway | 3 | 0.038735 | INHBB |
| Apoptosis | 3 | 0.049822 | CYCS |
| Apoptosis | 3 | 0.049822 | BCL2 |
| Apoptosis | 3 | 0.049822 | MAP3K14 |
