# Supplementary material for: Mir-34a Is Upregulated during Liver Regeneration in Rats and Is Associated with the Suppression of Hepatocyte Proliferation
Source: PLoS One. 2011 May 31;6(5):e20238. doi: 10.1371/journal.pone.0020238 (PMC3105003; doi:10.1371/journal.pone.0020238)
Supplement: Table S3 — Primers and sequences for qRT-PCR, siRNA transfection and plasmids construction. (DOCX) [file pone.0020238.s003.docx]

**Table S3.**

| Name | | **Sequences** |
| --- | --- | --- |
| miR-34a-rt^1^ | RT | 5'-GTCGTATCCAGTGCAGGGTCCGAGGTATTCGCACTGGATACGACAACAAC-3' |
|  | Sense | 5'-GTGCAGGGTCCGAGGT-3' |
|  | Anti-sense | 5'-CATGGCAGTGTCTTAGCTGGTT-3' |
| *u6-*rt | Sense | 5'- CTCGCTTCGGCAGCACA-3' |
|  | Anti-sense | 5'- AACGCTTCACGAATTTGCGT-3' |
| Actin-rt | Sense | 5'- TCGCACAATGACTCTGGAAG-3' |
|  | Anti-sense | 5'- CAGGGTCCACATTCAGGACT-3' |
| INHBB-rt | Sense | 5'-AGCAGACATCGCATCCGAAA-3' |
|  | Anti-sense | 5'-GGGTGCAATGATCCAGTCGTT-3' |
| Met-rt | Sense | 5'-ATTCATGGGCCGGCTCAA-3' |
|  | Anti-sense | 5'-CGAGAGAGCACCACCTGCAT-3' |
| INHBA-rt | Sense | 5'-AGAAAGTGGTAGATGCTCGGAAGA-3' |
|  | Anti-sense | 5'-ACAAGCAATCCGCACATCCA-3' |
| FAM siRNA control | Sense | 5'-UUCUCCGAACGUGUCACGUTT-3' |
|  | Anti-sense | 5'-ACGUGACACGUUCGGAGAATT-3' |
| INHBB siRNA | Sense | 5'-GGAGGUAUGUACUGUAAGUTT-3' |
|  | Anti-sense | 5'- ACUUACAGUACAUACCUCCTT -3' |
| Control siRNA | Sense | 5'-UUCUCCGAACGUGUCACGUTT -3' |
|  | Anti-sense | 5'-ACGUGACACGUUCGGAGAATT -3' |
| INHBB 3'-UTR | Sense | 5'-GCGTCTAGA CAGGGAGGCAGTGGTAGAG-3' |
|  | Anti-sense | 5'-GGGGCCGGCCTATGTGTCAGGCAAGGGCTCT-3' |
| Mu-INHBB 3'-UTR | Sense | 5'-ACACCTCATTTGAAAAAAAGTTATTTTTATAGCTGCA-3' |
|  | Anti-sense | 5'-TTTCTGACTAACCATGCAACTAGATCA-3' |
| p53 Binding Site 1 | Sense | 5'-CTTTCGCCCACCTTGACC-3' |
|  | Anti-sense | 5'-GGCCTAGTTCCTGCCTCCTT-3' |
| p53 Binding Site 2 | Sense | 5'-CAGGCATCCACCCGAACA-3' |
|  | Anti-sense | 5'-CAGCAGACAACGGGCAAAG-3' |

^1^rt: real-time PCR primer
